# On Void Formation during the Simulated Tensile Testing of Polymer–Filler Particle Composites

**DOI:** 10.1021/acs.macromol.6c00355

**Published:** 2026-07-21

**Authors:** John J. Karnes, Supun S. Mohottalalage, Amitesh Maiti, Andrew P. Saab, Todd H. Weisgraber

**Affiliations:** 4578 Lawrence Livermore National Laboratory 5000 East Ave., Livermore, California 94550, United States

## Abstract

We simulate model polymer composites composed of linear polymer strands and spherical, monodisperse filler particles (FP). These molecular dynamics simulations implement a coarse-grained, bead-spring force field, and we vary several formulation parameters to study their influences on material properties. These parameters include FP radius, FP volume fraction, temperature, and the polymer–polymer, FP–FP, and polymer–FP interaction potentials. Uniaxial extension of the simulation cells allows direct comparison of the mechanical reinforcement (or weakening) provided by the FP. We focus on the formation of microscopic voids during simulated tensile testing of glassy polymer composites and quantify how the characteristic spatial and morphological arrangement of these voids varies as a function of the interaction potentials used in the simulations. We discuss the implications for polymer composite design and formulation, specifically observing that an interaction scenario with a repulsive polymer–FP cross-interaction produces additional mechanical reinforcement above the glass transition temperature (*T*_*g*_) but is detrimental to performance below *T*_*g*_, at the FP sizes and volume fractions examined in this work.

## Graphical abstract

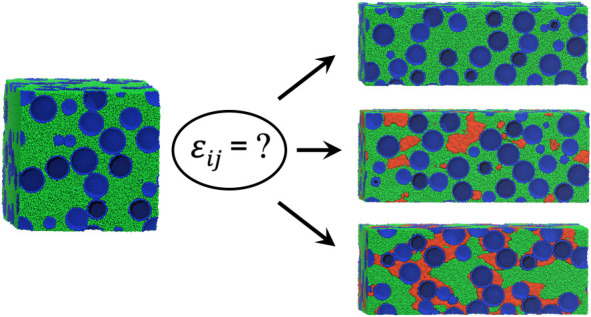

## Introduction

Polymers are a unique class of materials, defined by the chains of covalently bonded, repeating subunits that comprise them.^1,2^ Now commonplace, the “macromolecular hypothesis” of the material’s microscopic structure met strong resistance when first proposed by Staudinger in 1920.^3^ The chemical makeup of these subunits, length of the chains, and cross-linking and entanglement of the chains are a few of the tunable parameters that affect the properties of the resulting material. This parameter space allows for the design of polymers optimized for a wide range of properties, including tensile strength, thermal conductivity, electrical insulation, resistance to chemical degradation, and wetting. Further, polymer morphology can be tuned for more esoteric properties based on structure, including the synthesis of nanoporous materials with low areal densities that permit them to function as surrogates for high density gases.^4–6^ The ever expanding set of macroscopic properties that emerge from the interplay of microscopic interactions continues to inspire interest in polymers whenever a bespoke set of properties is needed for a new application.

Beyond the design and formulation of the polymer, the addition of distinct filler materials like carbon fibers or silica particles to the polymer matrix can be exploited to tune material properties outside the range achieved by reformulation of the polymer alone.^7–11^ The filler material’s geometry, chemistry, and interaction with the surrounding polymer matrix all affect the properties of the resulting composite, and these changes may be desirable or detrimental as compared to the neat polymer for a given application.^12^ In many applications filler particles (FP) are incorporated into a polymer matrix to enhance the mechanical performance of the material.^7,13^ FP size may vary from hundreds of μm to tens of nm, with particles having a size on the order of the polymer’s radius of gyration (*R*_*g*_) typically having the strongest effect. This approach has proven effective for many use cases, despite the ongoing debate regarding the origin of the enhancement.^14–18^

Lattice Monte Carlo studies have suggested that spherical FP with radii close to the polymer’s *R*_*g*_ provide mechanical reinforcement by facilitating the creation of transient polymer networks that bridge the FP, that this reinforcement is a strong function of the mean interparticle distance.^19^ Later experimental studies have proposed that mechanical reinforcement in composites is due to the formation of FP agglomerates and that bulk deformation causes the breakdown and subsequent reformation of these agglomerates.^20^ It has been suggested that the force driving this agglomeration increases linearly with FP radius, independent of any explicit interparticle attractive potential.^21^ More recent molecular dynamics simulations performed in the glassy state agree with earlier computational work, and suggest that mechanical enhancement by FP arise from the polymer bridging effect, even in the case of polymers well below the entanglement length.^15,22^ Deep understanding of these microscopic phenomena can assist the development of functional composite materials, particularly when balancing physical and economic drivers.

The vastness of polymer design space is a challenge onto itself due to the *formulate-fabricate-test* nature of experimental work. Due to practical limitations of chemical synthesis and formulation, thorough experimental parameter sweeps are both resource-intensive and often incomplete. Computer simulation is an alternative approach toward surveying this parameter space that can circumvent some of the challenges of an experimental campaign. All-atom, particle-based molecular dynamics simulations have demonstrated the ability to capture important properties of polymer melts and materials^23–26^ but similar studies of polymer composites require access to larger time and length scales, making all-atom simulations of these systems inaccessible with modern computational resources.^27–29^ Successful simulation of polymer composites must balance efforts to accelerate calculations while retaining the fidelity required by the specific problem. Coarse grained models reduce the cost of computer simulation by merging multiple atoms into a single interaction site. This approach reduces computational expense in two fundamental ways: First, reducing the number of particles in the system directly reduces the number of pairwise interactions that must be computed. Second, coarse-graining typically removes the highest frequency vibrations in a simulation. This allows the simulation to be performed with a longer time step since the maximum length of the time step is dictated by the fastest motion in the system.

Exploring polymer design space with computer simulation enables a major philosophical shift, where fundamental parameters like polymer chain length or interaction energies may be adjusted without having to first consider wet chemical synthesis or formulation; these challenges can be reserved for when promising candidate systems are identified. Similarly, coarse-grained simulations that implement well-validated models can provide molecular insight into the origins of the macroscopic properties that they model.^15,30^

In this work, we present a series of simulations that surveys parameters of interest to the polymer composite community: Filler particle (FP) size, FP volume fraction, polymer–FP interaction energy, and system temperature. We implement the well-traveled Kremer-Grest coarse-grained model^31^ to represent both the polymer matrix and the particles that constitute filler particles in the simulations. This broad survey is conducted within the confines of a single “universal” model design so that trends that span the various parameter sweeps are more clearly correlated with their microscopic origins.

## Computational Model and Methods

### Force Field and Model

Pairwise interactions are described by the Lennard-Jones (L-J) potential,

1 $$U_{ij}\left(r\right)=4\epsilon_{ij}\left[{\left(\frac{\sigma_{ij}}{r}\right)}^{12}-{\left(\frac{\sigma_{ij}}{r}\right)}^{6}\right]$$

where *r* is the distance between coarse grained particles *i* and *j*. To generalize our results, we employ the Lennard-Jones unit system and let ϵ = 1 for all possible interactions between polymer and filler particle bead types. All particle types are assigned a mass of 1 and have a diameter σ = 1. We use the finite extensible nonlinear elastic (FENE) potential to represent bonds between beads in linear polymer chains,

2 $$U_{b}\left(r\right)=-\frac{1}{2}k_{b}r_{\max}^{2}\ln\left[1-{\left(\frac{r}{r_{\max}}\right)}^{2}\right]+4\epsilon \left[{\left(\frac{\sigma }{r}\right)}^{12}-{\left(\frac{\sigma }{r}\right)}^{6}\right]$$

where the first term is attractive and contains the force constant *k*_*b*_ = 30 and *r*_max_ is the maximum bond length, 1.5σ. The second term is cutoff at a distance of *r* = 2^1/6^σ, the minimum of the 12–6 L-J potential, making it fully repulsive.^31^

This work considers both canonical, repulsive-only interactions, where the pairwise interaction in eq 1 is truncated at a distance of *r_c_* = 2^1/6^σ and shifted such that *U*(*r*_c_) = 0 (i.e. the Weeks-Chandler-Andersen (WCA) potential), and the case of attractive particles, where eq 1 is truncated (and shifted) at *r*_c_ = 1.75σ.^15,32^ We also simulate the cases of dissimilar interactions between the polymer and FP; where polymer–polymer and FP–FP interactions are repulsive-only and polymer–FP interactions are attractive and the inverse case where polymer–FP interactions are repulsive-only. All simulations are performed using the open-source LAMMPS codebase.^33^

Model filler particles (FP) are constructed by arranging K-G beads onto the surface of a sphere. To do so, we define the centers of the surface beads by constructing a Fibonacci Sphere of the desired radius *r* with 4π*r* ^2^ surface points. Each point defines the location of a K-G bead and one additional bead is placed at the center of the FP. Implementation of these composite hollow spheres is considerably less expensive than representing each FP with a single bead of radius *r* = 10 or *r* = 5, since the latter would dramatically increase the minimum cutoff distance for force calculations in the simulation. Each FP, consisting of 4π*r* ^2^ + 1 beads, is treated as a rigid body by assigning the beads constituting each FP a distinct molecule ID and using the LAMMPS function fix rigid. This function ensures that the FP remains precisely the same shape and size during the simulation. A given filler particle moves and rotates as a single entity during MD simulation. The total force and torque on each FP are computed as the sum of the forces and torques on the constituent beads.

### System Setup, Relaxation, and Mechanical Testing

Composite polymer–particle systems are prepared using our in-house code, which generates a low-density initial configuration based on the desired FP size, FP volume fraction ϕ, and polymer chain length. We impose a standard final polymer phase density of 0.87 beads/σ^3^ and a final box edge length of 97.31σ for all simulations. This set of parameters fully constrains the definition of a polymer–FP system. Initial configurations are prepared by randomly placing the appropriate number of FPs into a box with edge length defined so that the polymer phase will have a density of 0.01 beads/σ^3^. Polymer strands are then inserted using a self-avoiding random walk approach, with additional considerations taken to ensure that the polymer strands do not overlap the FPs. These low-density configurations are initially relaxed using a soft interaction potential to escape any high-energy states, followed by introduction of the desired L-J potential. Our relaxation approach is similar to that implemented by Riggleman and co-workers^15^ and consists of heating the simulation to *T* = 10, compression to the final polymer density of 0.87 beads/σ^3^, and cooling to simulation temperature over the course of ∼5 × 10^6^ MD steps with a time step τ = 0.002. For linear, fully flexible bead-spring polymers, as in this study, the entanglement length has been determined to be *N*_*e*_ ≈ 84. Thus, our chosen chain-length of *N* = 100 corresponds to a system of slightly entangled chains. The strand length and our equilibration and annealing cycles were selected as a conservative approach to ensure that the resulting polymer melts represent a relaxed configurations prepared within available computational resources. Figure 1 shows a representative FP, polymer strand, and relaxed simulation cell as implemented in this work.

**Figure 1:**
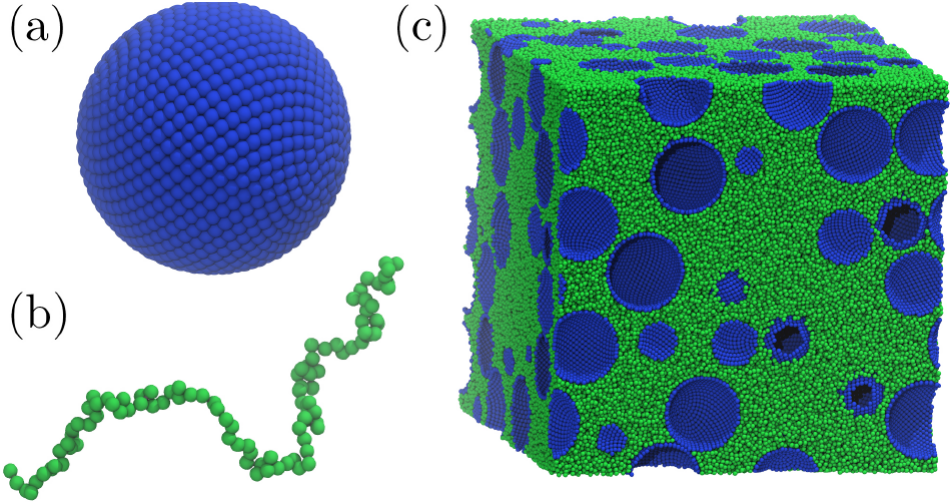
(a) Hollow Fibonacci spheres of K-G beads, example shown with radius = 10σ, represent the filler particles and (b) polymer strands of *N* = 100 K-G beads are prepared by a self-avoiding random walk. (c) Representative simulation cell with ϕ = 42.5% after high-temperature relaxation and compression to final density.

Simulated tensile testing is performed by the uniaxial extension of the cubic simulation box along a selected axis (e.g., *x*) at a constant engineering strain rate of ${\mathrm{\epsilon ̇}}_{0}=2\times 10^{-5}$ while the orthogonal periodic boundaries (*y* and *z* axes) are controlled by the Berendsen barostat and therefore allowed to change size while maintaining a constant pressure. To assess the rate-sensitivity of our glassy-regime results, we performed additional uniaxial extension simulations at half and double the baseline rate (1 × 10^–5^ and 4 × 10^–5^) on a representative LJ configuration at *T* = 0.3. Stress–strain response and void formation were largely insensitive to strain rate across this range (see Supporting Information). At the maximum applied strain (ϵ = 1.0), the elongated cell dimension reaches ∼195σ and the orthogonal dimensions contract under the Berendsen barostat while remaining substantially larger than the largest voids observed in any of the simulated systems (∼15σ). Periodic-image effects on the reported void distributions are therefore not expected. To verify isotropic behavior, each relaxed starting configuration was tested by independent elongations along the *x*, *y*, and *z* axes. All stress–strain data shown in this work represents the average of these three uniaxial extension simulations, with noise reduced by a sliding window average. To verify that the reported trends are robust to choice of starting configuration, we performed independent replicate simulations using three independent starting configurations for the representative state points underlying Figures 2 and 3. Inter-replicate variability is small compared to the differences observed in the parameter sweeps. All replicate data and analyses are provided in the Supporting Information. Our chosen strain rate ensures that systems either above *T*_*g*_ (*T* = 1.0 in this study) or modeled with the WCA potential are in the quasi-static regime, with a Deborah number *D*_*e*_ ≈ 0.2, representing equilibrium-like stress–strain response. In the case of an attractive potential and *T* = 0.3 we expect the material to be glassy and therefore a quasi-static uniaxial extension is computationally prohibitive. We recognize that some observed behavior and phenomena may be functions of strain rate *ϵ̇* and present findings with the most conservative, yet accessible, choice of *ϵ̇* and provide insight into both the composite material’s mechanical reinforcement and the origins of mechanical failure.

**Figure 2:**
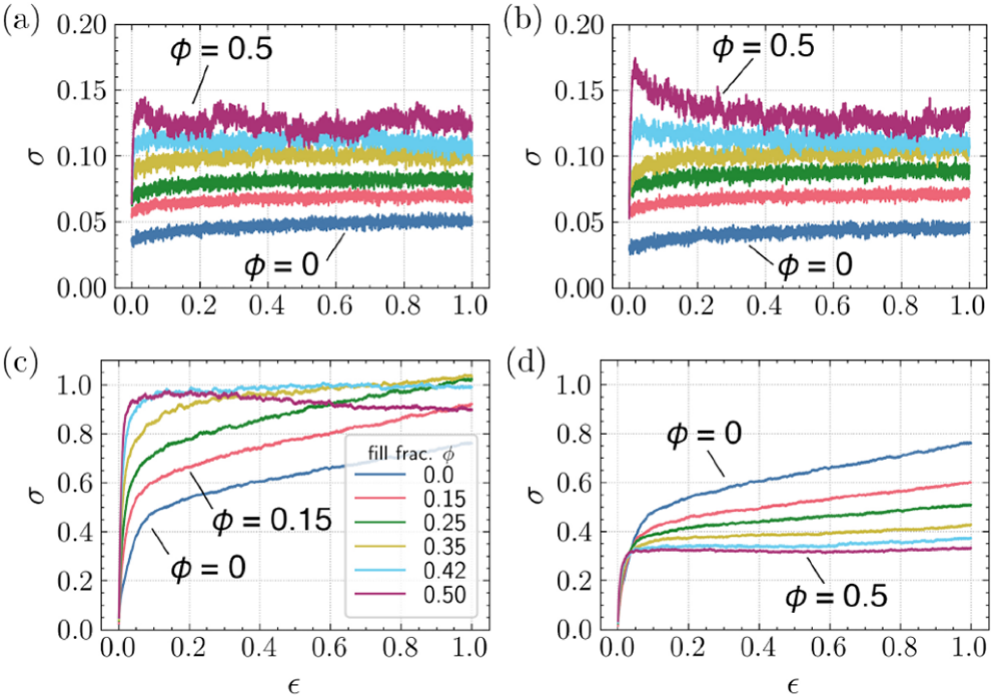
Simulated tensile testing results for polymer–filler composites for selected FP fill fraction ϕ. Panels (a) and (b) are above *T_g_* with WCA (panel (a)) and LJ (panel (b)) interactions implemented between all particle types. Panels (c) and (d) are at *T* = 0.3, in the glassy state. In (c) all pairwise interactions are slightly attractive (LJ), while in (d) like interactions (ϵ*_ii_*) are LJ and mixed interactions (ϵ*_ij_*, i.e., polymer–FP) are described by the repulsive-only WCA potential. All FP have a radius of 10σ.

**Figure 3:**
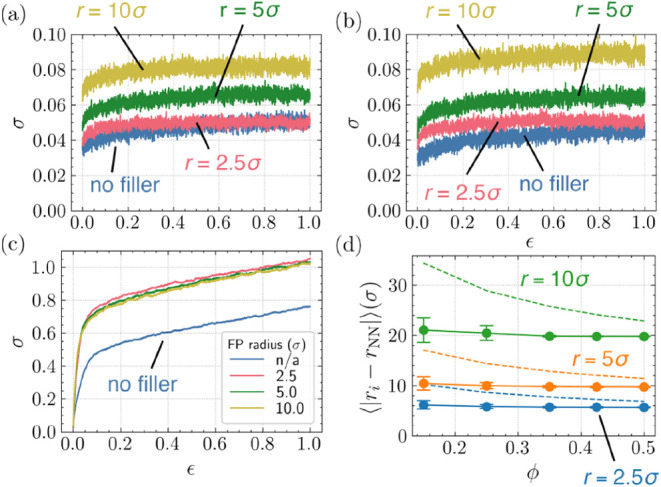
Simulated tensile testing as a function of filler particle radius. ϕ = 0.25 for all polymer composites. The top row of panels shows results at *T* = 1.0 for the (a) WCA and (b) LJ interaction potentials. Panel (c) shows stress–strain curves for the LJ system at *T* = 0.3. In (d), FP nearest neighbor distances from *T* = 1.0 WCA simulations are represented by solid curves and error bars represent one standard deviation. Values for the corresponding ideal case of equidistant FP are shown as dashed curves of the same color.

As mentioned earlier, the phase space made available by the simulation protocol introduced in this work is vast. Accordingly, we focus on a subset of parameters that are most relevant to our related work and examine all resulting combinations.^34,35^ For all simulations, K-G beads have a diameter of 1σ and the number of K-G beads per polymer chain is set to *N* = 100. In all simulations with filler particles the FP are monodisperse Fibonacci spheres with a radius of 2.5, 5.0, or 10.0σ and filler volume fractions (ϕ) ranging from 15% to 50%. Real filler materials are typically polydisperse, and the effects of FP size distributions on packing, agglomeration, and mechanical properties are themselves worthwhile subjects of study. We isolate the role of FP size in this work by comparing these three discrete FP radii (2.5σ, 5σ, and 10σ) across otherwise-equivalent monodisperse systems, leaving polydispersity-driven effects for future investigation. For pure linear-chain polymers modeled by canonical K-G, *T*_*g*_ ≈ 0.39 and is known to increase with increasing filler content. System temperatures of *T* = 1.0 and *T* = 0.3 are studied to capture both the canonical K-G model and the low-temperature (glassy) regime recently studied by Riggleman and coworkers.^15^ In filled composites the glass transition temperature generally shifts relative to the neat polymer, with the direction and magnitude depending on the polymer–filler interfacial chemistry: attractive interfaces typically elevate *T*_*g*_ through reduced mobility of polymer adjacent to the filler surface, while weak or repulsive interfaces can leave *T_g_* unchanged or shift it downward. We did not measure *T*_*g*_ independently for each composite system in this study; rather, our temperature choices are intended to bracket *T*_*g*_ conservatively across all interaction scenarios examined. *T* = 0.3 lies well below the neat polymer *T*_*g*_ ≈ 0.4 and is therefore below any plausible composite *T*_*g*_ for the systems studied, while *T* = 1.0 lies well above. Polymers do not exhibit a glass transition in the canonical K-G model or when interaction strengths are less than 0.4 *k*_*B*_ *T*, so the WCA-only composites are not in a glassy state at either temperature.

In this work, interaction potentials ϵ may be either fully repulsive, using the Weeks–Chandler–Andersen (WCA) formulation, or allow for interparticle attraction by extending the cutoff of this interaction to 1.75σ. Polymer–polymer, polymer–filler, and filler–filler interactions are varied, resulting in four scenarios. In this work we refer to the repulsive only interaction as “WCA” and to the attractive potential (i.e., with *r*_cutoff_ = 1.75σ) as “LJ”. The four scenarios correspond to distinct physical situations in real composite materials:

1 **WCA**: all interactions repulsive-only. The reference case with no attractive interactions; no glass transition occurs and the polymer behaves athermally. Useful for isolating entropic effects.
2 **LJ**: all interactions attractive. Models a composite in which the polymer–FP interfacial chemistry is similar to the bulk polymer cohesion. Below *T*_*g*_ the system is glassy throughout.
3 **interLJ**: like-pair interactions repulsive (*WCA*), cross-pair (polymer–FP) interaction attractive (*LJ*). Included to complete the permutation of attractive/repulsive like- and cross-pair combinations, enabling independent assessment of the role of each interaction type. Physically corresponds to a system in which the polymer–FP interface provides the dominant cohesion, with neither bulk phase being self-cohesive.
4 **interWCA**: like-pair interactions attractive (*LJ*), cross-pair (polymer–FP) interaction repulsive-only. Models a chemically passivated filler in a cohesive polymer matrix, e.g., methyl-terminated fumed silica in PDMS, where surface functionalization is used to suppress the otherwise-attractive silica–PDMS interaction. The polymer phase remains cohesive (and can become glassy at low *T*) while the FP–polymer interface is the weakest interaction in the system.

Table 1 also summarizes simulation settings and the parameter space explored in this work.

**Table 1:** Listing of Simulation Parameters

Parameter	Symbol	Value(s)
Box edge length, σ	*l*_0_	97.31
Chain length	*N*	100
Polymer bulk density, σ^–3^	ρ	0.87
Same-type interaction^a^	ϵ_*ii*_	WCA, LJ
Cross-type interaction^b^	ϵ_*ij*_	WCA, LJ
Temperature	*T*	0.3, 1.0
Filler vol %	ϕ	15, 25, 35, 42.5, 50

a Polymer–polymer and FP–FP.

b Polymer–FP.

The in-house code used to generate polymer-composite molecular dynamics starting configurations and associated LAMMPS input files is freely available at https://github.com/karnes/K-G-and-Meatballs. Complementary characterization of simulated polymer composites prepared using this software and protocol is provided in our recent work.^36^

## Results and Discussion

Figure 2 is an overview of simulated tensile testing as a function of filler particle (FP) volume fraction, ϕ. In panels (a–d) the FP volume percent ranges from 0%(neat polymer) < ϕ < 50%. Stress–strain curves in all panels share the same ϕ vs color scheme as defined in the panel (c) legend. For all cases in Figure 2 the radius of the filler particles is 10σ. We note that, in practice, FP packing above 50 volume percent leads to numerical instabilities during the high-temperature compression step of our relaxation protocol, specifically instances where FENE bond lengths exceed the maximum length, *r* > *r*_max_ (see eq 2). This instability occurs near the lowest known geometric packing density at which jammed configurations of monodisperse hard spheres can exist, $\phi =\pi \sqrt{2}/9\approx 0.4937$ .^37^ Geometric proximity to this packing bound does not by itself imply mechanical jamming of the filler network, which requires additional structural conditions examined later in this work. This instability can be circumvented by using a harmonic potential to describe bonds within the polymer chain and this approach has been applied to simulate packing fractions up to 60 vol %.^15^ For reference, we note that the theoretical limit of close packed spheres is $\phi =\frac{\pi }{3\sqrt{2}}\approx 0.7405$ .^38^ The FENE potential, as implemented in this work, limits bond elongation so that polymer chain crossover is not permitted, even with very high local forces. We note that this chain crossover during equilibration and relaxation is not problematic in the study of polymers below the entanglement length, but for long polymers chain crossover directly affects entanglement statistics.

Figure 2a shows the stress–strain responses for the canonical K-G model, with the WCA interaction potential and a temperature of *T* = 1.0, as a function of ϕ. The stress increases with increasing ϕ for all packing fractions, indicative of the mechanical reinforcement provided by the filler particles. At the highest fill fraction, 0.5, stress strain response is less smooth, suggesting that the simulation box moves through “stick–slip” configurations during the uniaxial extension. Panel (b) shows the response when the attractive LJ potential is implemented. At *T* = 1.0 stress–strain response for the WCA and LJ potentials is rather similar, with somewhat increased stress near the early elastic region of the high fill fraction cases. These results confirm that the addition of a potential well that corresponds to ϵ = 1 has little impact on the mechanical response of the polymer composite at *T* = 1.

Introduction of this attractive potential results in the polymer phase having a glass transition temperature of *T*_*g*_ ≈ 0.4.^15^ Panel (c) shows the stress–strain response for the LJ system at *T* = 0.3, in the glassy regime. Simulated tensile testing below *T*_*g*_ resulted in stress curves about an order of magnitude larger than the *T* = 1.0 cases, with a similar increase in stress with increasing FP packing. At higher packing fractions, yield-like behavior emerges as the polymer domains become smaller and the average FP–FP distances decrease. We note that the glassy regime is only accessible at reduced temperatures when the attractive LJ potential (ϵ = 1) is implemented; polymers do not undergo a glass transition in the canonical K-G model or when interaction strengths are less than 0.4 *k*_*B*_ *T*.^39,40^

Panels (a–c) all exhibit increasing stress–strain with increasing ϕ, regardless of whether the simulation is executed with repulsive-only (WCA) or attractive interparticle potentials (LJ). In panel (d) we mix these interactions to simulate a change in the composite’s formulation: like-particle interactions (e.g., polymer–polymer) are LJ but mixed particle interactions, polymer–FP, are represented by the WCA potential to represent FP with chemically passivated surfaces. This reformulation reverses the trend seen in panels (a–c), where the noninteracting FP effectively lubricate the material and result in a monotonic decrease in stress–strain response with increasing fill fraction.

Figure 3 shows the effect of varying FP radius on tensile response. For these simulations, ϕ = 0.25 is selected as a representative intermediate value of fill fraction. FP radii of 2.5, 5.0, and 10.0σ and the neat polymer system are compared. These values were selected to span the radius of gyration of the *N* = 100 Kremer-Grest polymers, about 5σ in these studies. Experimental studies of FP much larger than *R*_*g*_ observe that smaller FP provide better reinforcement.^41^ Figure 2 is similar to the survey of fill fraction ϕ, presenting the WCA and LJ potentials at *T* = 1.0 in panels (a) and (b). At *T* = 1.0 stress–strain response increases slightly with increasing FP size, consistent for both force fields. We explain this enhancement by referencing that mechanical reinforcement emerges from the creation of transient polymer networks that bridge the FP. With increased radius of curvature, the bridging networks in larger radius systems are more robust and contribute more enhancement than is provided by the additional number of particles in a smaller FP radius system. When below *T*_*g*_, as in panel (c), the LJ system shows an enhanced mechanical response that is relatively agnostic to FP size. In the glassy state, general rearrangement of the polymer itself during the deformation is much more energetically intensive than the local reordering around the filler particles as a function of FP radius. Although the stress differences are relatively small in the glassy state, systems with larger particle radius provide slightly less enhancement, suggesting that rearranging the glassy polymer around a greater number of smaller FP is more energetically expensive than effects due to local transient reinforcement networks. The small impact of FP size, particularly when within a stiffer polymer, agrees with experimental studies of cross-linked acrylonitrile butadiene rubber (NBR), where the authors found that increasing ϕ enhanced mechanical reinforcement but varying FP diameter from 15 to 28 nm had no significant effect on stress–strain response for a given fill fraction ϕ.^42^

In experimental formulations filler materials similar to those simulated in this work are known to self-assemble into larger, tightly bound agglomerates, often with dendritic morphology.^9^ We did not implement forces to explicitly encourage or discourage the agglomeration of filler particles but note that this behavior was observed in all simulations. Figure 3d summarizes the global drive toward agglomeration observed in our simulations. The average FP–FP nearest neighbor distance, ⟨|*r*_*i*_ – *r*_NN_|⟩, where *r*_*i*_ is the position of an FP of interest and *r*_NN_ is the position of its nearest neighbor FP, is plotted versus ϕ for selected FP radii. For reference, we include dashed curves which represent the FP–FP separation distance for the ideal case of maximum separation, where FP are equidistant from each other within the polymer. For all cases, the value of ⟨|*r*_*i*_ – *r*_NN_|⟩ is close to twice the FP radius, deviating significantly from the case of equal spacing. The pure repulsive WCA case is shown in Figure 3d, highlighting the entropic drive toward particle agglomeration. We reserve further investigation of the effects of FP spacing, FP–FP bonding, and FP agglomerate morphology for future work.

Figure 4 further considers the effects of interaction potential on the systems’ stress–strain response. In panel (a) the temperature was held at *T* = 1.0 and both the neat polymer and 25 FP volume % configurations were considered, ϕ = 0, 0.25. As observed earlier, the difference between WCA and LJ interaction potentials is very small for the neat polymer and ϕ = 0.25 systems above *T*_*g*_. Mechanical reinforcement is observed with the addition of FP. In this figure we also introduce mixed interaction potentials to simulate dissimilar FP and polymer chemistries, which is to be expected in most laboratory formulations. For this approach the first of the two cases (introduced but not named in Figure 2d) implements LJ interactions between like particles and WCA interaction between dissimilar particles: Polymer–polymer interactions and FP–FP interactions have an attractive LJ potential with ϵ = 1 and polymer–FP interactions use the WCA potential, which contains only the repulsive part of the Lennard-Jones potential. We refer to this case as *interWCA* to indicate that the interactions between dissimilar particle types are governed by the WCA potential. The second case reverses the interactions: polymer–polymer and FP–FP use the WCA potential and polymer–FP interactions are the attractive LJ. We refer to this case as *interLJ*.

**Figure 4:**
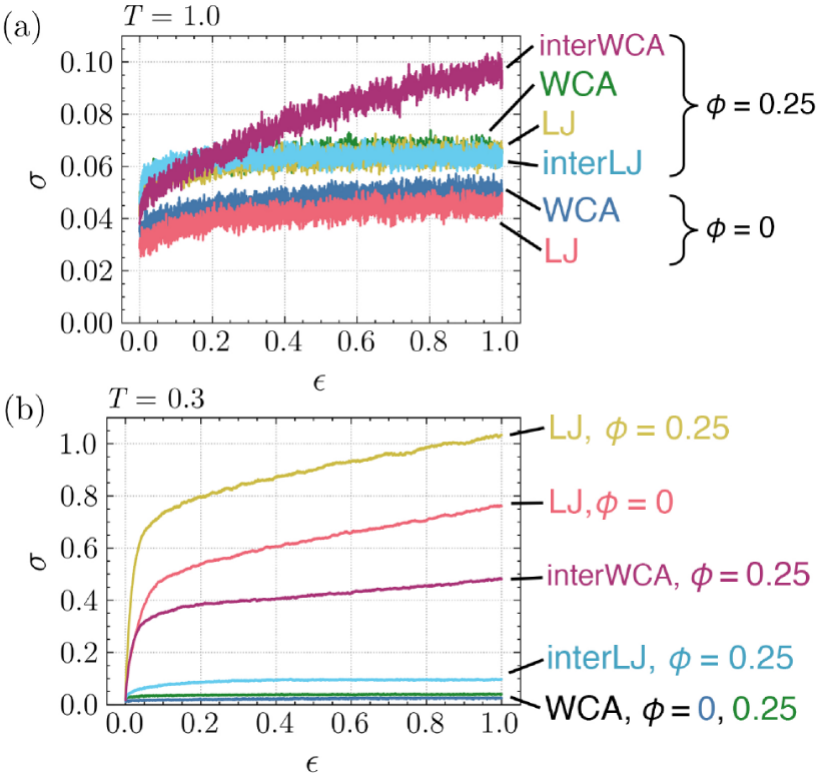
Simulated stress–strain response as a function of system interaction potential for (a) *T* = 1.0 and (b) *T* = 0.3 conditions. For all composite systems FP radius *r* = 5σ.

The *interLJ* system behaves similarly to the pure WCA and LJ cases in Figure 4a, which indicates that introducing an attraction between polymer and FP does not significantly perturb system behavior at *T* = 1.0 since both systems are above *T*_*g*_. Notably, the *interWCA* potential does introduce new behavior, showing increasing stress with deformation. At the microscopic level, deformation of the *interWCA* system appears to create behavior analogous to the hydrophobic effect, where disruption of the cohesive polymer phase by filler particles (which are similarly attracted to other filler particles) is energetically unfavorable in addition to being entropically unfavorable as in the WCA and LJ cases. For the specific case examined here, the *interWCA* scenario, which models a chemically passivated FP surface against a cohesive polymer matrix, enhances the mechanical reinforcement provided by the filler above *T*_*g*_ relative to the other three interaction scenarios.

Tensile responses become more complex at *T* = 0.3 in Figure 4b. Both of the WCA systems, ϕ = 0 and ϕ = 0.25, behave similarly to *T* = 1.0 since these configurations are not in the glassy state. The interaction potentials used in the *interLJ* system are similar to the WCA ϕ = 0.25 system but introduce attraction between FP and polymer, resulting in a noticeably higher stress during deformation. This additional stress is notable in the context of designing composite materials since it represents both preferential polymer–filler interactions and the tethering of the polymer to the FPs. These interactions, although weak, reduce polymer mobility when near the FPs and result in noticeable mechanical enhancement. The increased stress due to the *interLJ* potential is a small perturbation compared to the neat polymer at *T* = 0.3 with LJ interaction. The tensile response of the neat glassy polymer is a convenient baseline for comparing the final two composite systems, which add 25 vol % filler to the system. Addition of filler particles with the LJ potential mechanically reinforces the system, with behavior seen in Figure 2c. In the *interWCA* case, the FP–polymer interface is governed by the repulsive-only WCA potential and the FPs effectively lubricate motion in the glassy system, resulting in a more easily deformed system: not the mechanical enhancement expected by the addition of FPs. This behavior is relevant to the design of composite systems that may be exposed to temperatures below the polymer *T*_*g*_, though direct quantitative extrapolation to specific industrial formulations would require validation against chemically specific models and the broader strain-rate range relevant to real-world loading conditions.

Figure 2 through 4 capture the full simulated tensile tests and primarily describe the yield stresses of these materials. We also consider the materials’ elastic response by calculating the slope of the initial linear region, using a conservative cutoff of 0.1% strain for all cases. Selected Young’s modulus (elastic modulus, *E*) values are shown in Figure 5 for high temperature (top row) and low temperature (bottom row) simulations of the *LJ* and *interWCA* potential composites. Error bars indicate the *R* ^2^ values of the linear fits and are notably larger for the noisier high temperature cases. At *T* = 1.0 there is a general trend of increasing modulus with ϕ for both potentials and all FP radii. The notably high value of *E* for the ϕ = 0.5, *r* = 10σ simulation in panel (a) agrees with the behavior in Figure 2b and reflects the compact, near-percolating filler agglomerate that develops at this fill fraction (see contact network analysis below). The low temperature cases in panels (c) and (d) show similar trends, *E* monotonically increasing with ϕ but less scatter than observed in the *T* = 1.0 simulations. *E* also increases with increasing FP radius *r* for most simulations.

**Figure 5:**
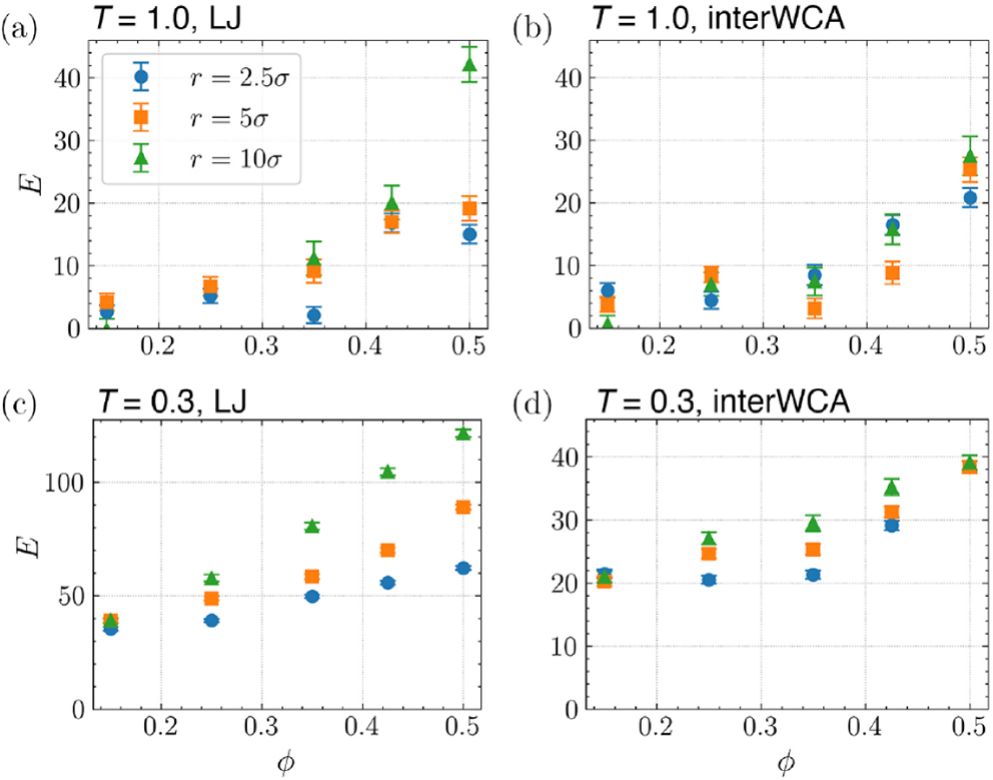
Young’s modulus (*E*) trends with increasing particle fill fraction ϕ for (a) LJ and (b) interWCA composites at *T* = 1.0 and (c) LJ and (d) interWCA composites at *T* = 0.3.

In the *LJ* simulations, panels (a) and (c), the large increase when below *T*_*g*_ is expected behavior and values agree with tensile tests of similar glassy composites.^15^ Regarding the *interWCA* composite, we emphasize that panels (a), (b), and (d) of Figure 5 have the same *y*-axis range. At *T* = 0.3, the low *ϕ interWCA* systems exhibit a much larger modulus than the corresponding high temperature cases. With increasing ϕ, the differences between the systems’ *E* values decrease but do not converge before the simulation accessibility limit at ϕ  ≈  0.5. This behavior is consistent for all FP radii and indicates that the FP–polymer WCA interaction “lubricates” the uniaxial extension at *T* = 0.3 by an amount that increases with ϕ. Here we note that these two interaction potentials, *LJ* and *interWCA*, are of particular interest to polymer composite design since they simulate the effect of adding a chemical passivation layer to the filler particle, analogous to functionalizing fumed silica particles with terminal methyl groups for silicone formulations.^34,35^

To directly characterize the structural origin of the high-ϕ stress–strain response, we constructed contact networks from the FP centers in each configuration and computed three metrics: the mean coordination number *z*, the percolation order parameter $S=\left|C_{\max}\right|/N$ (the fraction of FP belonging to the largest connected cluster), and the cluster susceptibility χ, which peaks at the geometric percolation threshold ϕ_*c*_. Two FPs are defined as in contact when their center-to-center distance satisfies *d*_*ij*_ ≤ 2*R* + δ, with the contact shell δ examined at three physically motivated values spanning the LJ potential minimum to the polymer *R*_*g*_. Full methodology, including PBC-aware percolation testing, is provided in the Supporting Information.

Across the ϕ-series, χ peaks consistently near ϕ_*c*_ ≈ 0.35, locating the percolation threshold, while *S* rises sharply and reaches ≥0.98 at ϕ = 0.5. Essentially all FPs reside in a single connected cluster. However, *z* remains below the isostatic value (*z*_iso_ = 6 for frictionless spheres) at all conditions, reaching only ∼3.5–5.3 at the highest ϕ depending on δ. Spanning percolation across the simulation cell is not observed at any condition, consistent with the compact agglomeration documented in Figure 3d. The high-ϕ contact network is therefore best described as a near-complete but subisostatic compact agglomerate rather than a rigid jammed backbone; the stick–slip stress response in Figure 2a at ϕ = 0.5 corresponds to cooperative rearrangement of this dense cluster during deformation. At intermediate ϕ = 0.25 with the LJ potential, *S* decreases from 0.93 (equilibrium) to 0.48 (100% strain), evidencing attractive cluster breakup during extension, while the WCA system shows the opposite trend.

Beyond mechanical stress–strain response, the potential for material failure must be considered when designing or formulating composite materials. In this and earlier work, inspection of MD configurations reveals the emergence of voids within the deformed materials. Figure 6 shows simulation snapshots acquired at *T* = 0.3 and 100% strain for the ϕ = 0.425 system and includes cases where significant delamination and voids have formed within the composite. Each snapshot is a 2-D slice of the simulation cell, parallel to the axis of elongation. Polymer beads are green, FP particles are blue and the hollows within the rigid FP Fibonacci spheres are left uncolored. Panel (a) was obtained from the WCA system and no voidspace within the polymer phase is observed. The LJ potential was implemented in panel (b) and the glassy polymer composite has a noticeable population of voids within the polymer phase. These voids are located both adjacent to the FPs and isolated within the polymer. This interfacial/intrapolymer distribution is reasonable and anticipated since the polymer–polymer and polymer–FP interaction potentials are identical in this simulation. Panel (c) shows the case of *interLJ*, where the polymer–polymer interaction is not attractive but the polymer–FP interaction is. No voids appear in this case; the WCA polymer phase is well above its *T*_*g*_ and the small glassy domains at the polymer/FP interface do not introduce a noticeable morphological perturbation during the simulated tensile testing. In panel (d) voids are present and appear to be exclusively located at the polymer/FP interfaces. The *interWCA* potential implemented in panel (d) consists of attractive polymer–polymer interactions, and therefore a glassy phase at *T* = 0.3. The polymer–FP interactions are WCA, which includes only a repulsive component, thus making the polymer/FP interface a logical point of origin for delamination to occur during mechanical deformation. The snapshots in Figure 6 are representative of system behavior at *T* = 0.3, but this mode of analysis is inherently qualitative. In the following sections we develop quantitative measures of void size distributions, via pore size analysis, and of void spatial localization relative to the filler particles, via the voidspace distribution function *g*_*v*_(*r*), to characterize the failure modes systematically across the parameter space.

**Figure 6:**
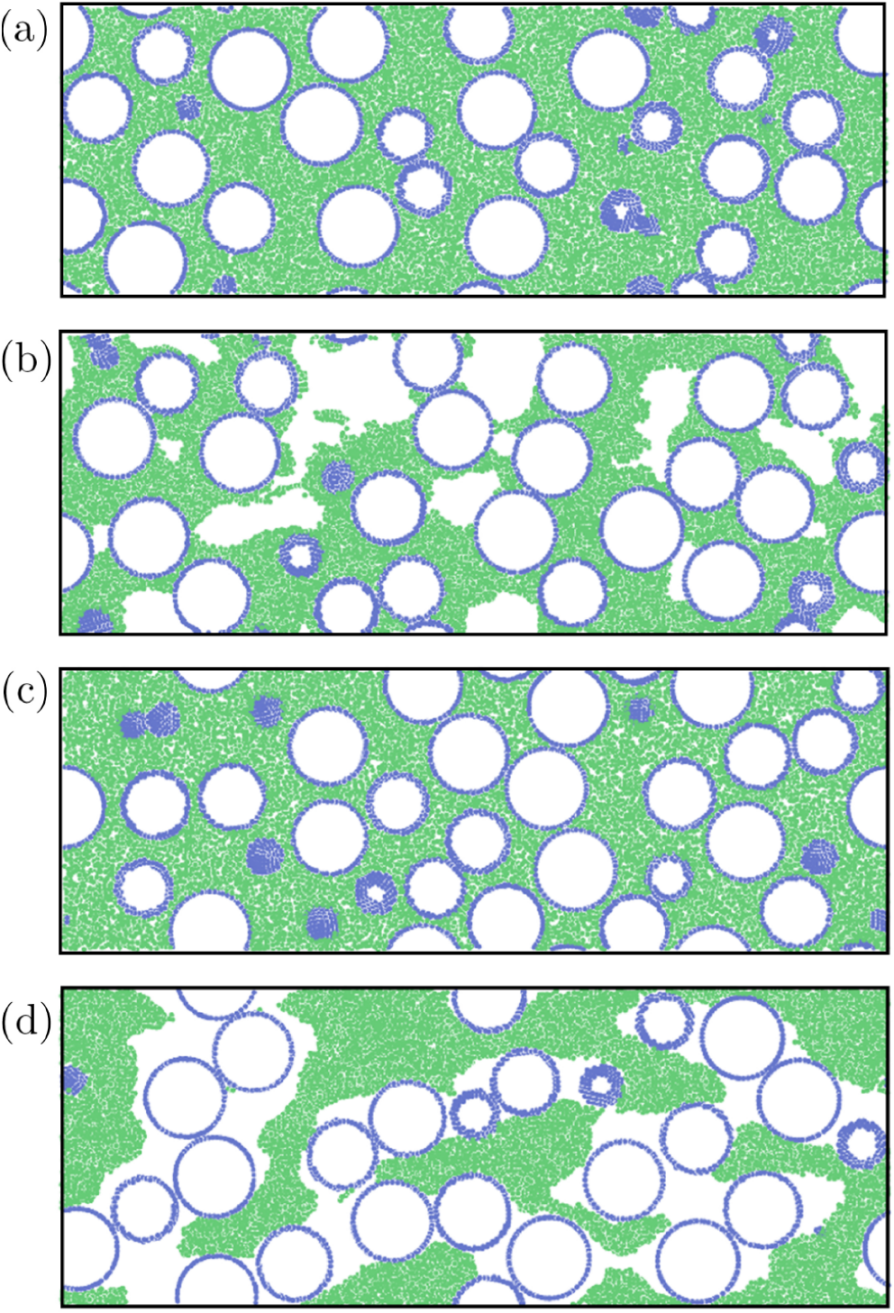
Simulation snapshots of 100% elongated polymer composites. Each snapshot is a 2-D slice parallel to the axis of uniaxial extension. The respective interaction potentials are (a) WCA, (b) LJ, (c) *interLJ*, and (d) *interWCA*.

Hagita and coworkers observed the formation of voids during simulated tensile testing of polymer composites at *T* = 1.0, well above *T*_*g*_.^43,44^ In our study no void formation was observed at *T* = 1.0 for any combination of interaction potentials and no voids were formed for systems that do not exhibit a glass transition (i.e., WCA, ϵ  <  0.4). We attribute this discrepancy to the difference in strain rate, 1.6 × 10^–3^ versus 2 × 10^–5^ in this work, a difference of about 2 orders of magnitude. The authors were aware of potential dependence on strain rate and noted hysteresis after elongation. The selection of *ϵ̇* in simulated tensile testing is largely influenced by available compute resources. While advances in computational power now permit simulations at quasi-static strain rates, we emphasize that some physical insights revealed by the work of Hagita and coworkers suggest the same design principles that arise from the present work.

Beyond the differences in strain rate considered above, we performed dedicated rate-sensitivity tests on a representative LJ configuration at *T* = 0.3 (see Supporting Information). The results indicate that void locations at ϵ = 1.0 are largely reproducible across a 4× range in *ϵ̇* when starting from the same initial configuration. This suggests that the spatial pattern of cavitation is determined primarily by features of the initial configuration rather than by rate-dependent kinetics, an observation worthy of dedicated future investigation.

We use PoreBlazer 4.0 ^45^ to quantify the sizes of the voids formed during simulated tensile testing. In brief, PoreBlazer computes a Pore Size Distribution (PSD) by selecting random points within the simulation that do not overlap the K-G beads. Each point is the center of a sphere and assigned a maximum diameter possible without overlapping any of the beads within the simulation. We refer the interested reader to the original text for a full description of the PSD algorithm.^45^

One nonstandard modification to the PoreBlazer input was required for our composite systems. Because the filler particles are constructed as hollow Fibonacci spheres of K-G beads (Figure 1a), a direct application of PoreBlazer would identify the interior of each FP as accessible pore space, since probe spheres can be inserted at the FP interior without overlapping any of the surface beads. To prevent this artifact, we set the effective van der Waals radius of each FP center bead equal to the FP radius itself, so that the center bead alone excludes probe insertions throughout the entire FP interior. This modification affects only the PoreBlazer input configurations and does not alter the underlying MD simulation.

Figure 7a shows PSDs which correspond to the snapshots in Figure 6b and d, with ϕ = 0.5 and FP radius *r* = 10σ for both cases. For the *interWCA* potential, where void formation initiates at the polymer/FP interface, the pore sizes are generally smaller than in the LJ simulation, where voids form at any given location. This behavior is consistent for other FP sizes as shown in Figure 7b. These simulations are similar to panel (a), with ϕ = 0.5 but a smaller FP radius of *r* = 5σ. For both sizes of FP, the average void radius is approximately equal to half of the FP radius for the *interWCA* potential and slightly less than the FP radius for the LJ potential. The voids may appear larger in panel (d) of Figure 6 than in panel (b) but the actual sizes are smaller since, in most cases, filler particles are located within the voidspaces.

**Figure 7:**
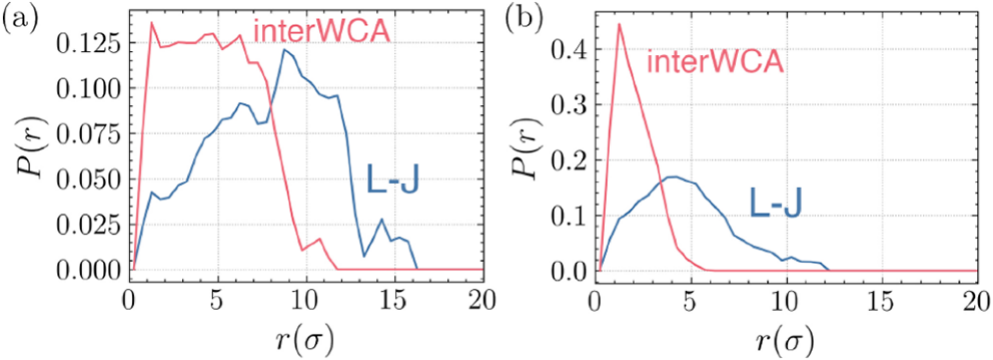
Pore distribution functions quantify the voids that emerge during simulated tensile testing of the polymer composites. Panel (a) shows the case of *r* = 10σ FP, (b) shows the distributions for *r* = 5σ.

PSDs are useful for quantitatively describing the size of the voids within the polymer composites but they do not provide information regarding the spatial location of these voids. We also note that PSDs based on maximal inscribed spheres are conservative measures of characteristic size for anisotropic voids: a single sphere inscribed within an elongated void reports the shortest principal dimension and underestimates the longest. Voids formed during uniaxial extension are expected to develop some anisotropy along the strain axis, so the absolute pore sizes reported in Figures 7 and 10a,b should be interpreted as lower bounds on true void extent but comparative trends across interaction potentials and fill fractions are preserved under this conservative reading.

A cursory inspection of Figure 6b and d suggests that voids are more likely to form at the polymer/FP interface in the *interWCA* composite than in the *LJ* system during uniaxial extension. To quantify this behavior, we developed an approach based on the standard pair distribution function *g*_*ij*_(*r*) calculation where, in this work, *i* is the center point of the filler particles and *j* represents an arbitrary point located within the voidspace. To implement, we randomly inserted 1σ diameter particles of a new LAMMPS type, dissimilar to both the FP and polymer beads, into the elongated configuration until all voidspace was filled with these “void” particles. This set of void particles serves to discretize the voids and enable quantitative description of their position relative to the FP by familiar means. From here, the calculation of a “voidspace” distribution function, *g*_*v*_(*r*), is straightforward,

3 $$g_{v}\left(r\right)=\frac{V_{\mathrm{cell}}}{N_{\mathrm{VP}}N_{\mathrm{FP}}4\pi r^{2}\Delta r}\overset{N_{\mathrm{VP}}}{\underset{i=1}{\sum }}\overset{N_{\mathrm{FP}}}{\underset{j=1}{\sum }}\left\langle \delta (\right|r_{i}-r_{j}\left|-r)\right\rangle$$

where VP and FP represent the void particles and centerpoints of the filler particles. Equation 3 is implemented using the LAMMPS compute rdf command with the FP centers as reference particles and the dummy void particles as targets. The normalization in eq 3 follows the standard pair distribution function convention, with the shell-volume factor (4π*r* ^2^Δ*r*) and reference density (*N*_VP_/*V*_cell_) ensuring that *g*_*v*_(*r*) asymptotes to 1 at large *r* for spatially uniform distributions. Peak amplitudes therefore represent the local enhancement of voidspace density relative to the system-wide reference density. A complete description of voidspace distribution function algorithm and its implementation is included in the Supporting Information.

Figure 8 shows the voidspace distribution functions for ϕ = 0.5 and FP radius *r* = 10σ. The region 0 < *r* < 10σ where *g*_*v*_(*r*) ≈ 0 corresponds with the volume excluded by the filler particles. The sharp peak in the *interWCA* curve at *r* = 11σ and associated shoulder correspond with a large population of voidspace adjacent to the FP particles’ surfaces. This is contrasted by the LJ curve, which shows a small shoulder peak at the FP surface followed by a broad peak centered at about 1.5 × the FP radius.

**Figure 8:**
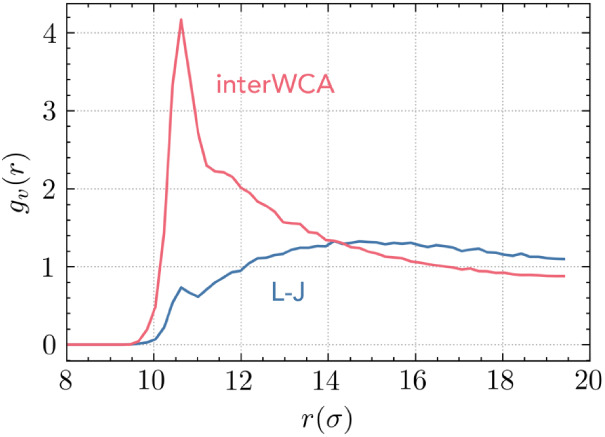
Voidspace distribution functions *g_v_* (*r*) quantify the location of voids relative to the filler particle centers for the LJ and *interWCA* potential simulations.

Figure 9a extends this analysis for different FP radii and confirms that the behavior is consistent. For all FP sizes surveyed in this work the weaker polymer–FP interaction in the *interWCA* potential results in more pronounced delamination at the FP interface than in the LJ case, where the attractive potential is constant for all pairwise species interactions. Panel (b) considers the void distribution function *g*_*v*_(*r*) for a range of fill fractions ϕ = 0.35, 0.425, and 0.5 and FP radius 10σ. The voids formed during simulated uniaxial extension are located at the FP–polymer interface for all *interWCA* cases as indicated by the sharp peaks at *r* = 10σ. For the *LJ* simulations the distribution function is more broad, with a relatively small peak at 10σ, indicating that the origin of each delamination is largely agnostic to the presence of FP–polymer interfaces. For all ϕ the respective curves for the *interWCA* and *LJ* cases look very similar to first approximation. The peak heights for *interWCA* increase with decreasing ϕ, which we attribute to more bridging of the voids between FP and increased numbers of local FP–FP neighbors reducing the magnitude of *g*_*v*_(*r*). A similar phenomena is seen in the *LJ* case, where the curves increase with decreasing ϕ since the lower packing fractions permit the formation of larger voids.

**Figure 9:**
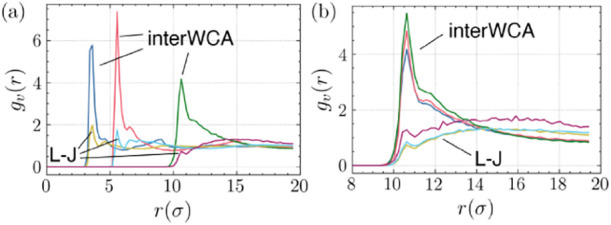
Voidspace distribution functions for (a) different FP radii and (b) fill fractions ϕ reveal the consistent spatial arrangement of cavitation and delamination during simulated uniaxial extension of the polymer composites.

The insight provided by Figure 4a, that FP with chemically passivated surfaces provide enhanced mechanical reinforcement above *T*_*g*_, must be tempered by the results of our voidspace studies. Reducing the interaction potential between FP and polymer may provide enhanced mechanical reinforcement above *T*_*g*_, but this formulation also introduces a distinct failure mode when system temperature approaches the polymer’s *T*_*g*_, a failure mode which also results in a dramatically reduced stress–strain response compared to the homogeneous attractive *LJ* potential, observed in Figure 4b. This assembled knowledge enhances the importance of Figure 2d, which shows that the loss of mechanical reinforcement that accompanies the *interWCA* potential below *T*_*g*_ increases with increasing fill fraction ϕ.

Lastly, we also consider the evolution of voids during simulated tensile testing of the *LJ* and *interWCA* systems. Figure 10 shows pore size distributions and voidspace distribution functions for these two systems as a function of applied engineering strain for the ϕ = 0.5, *r* = 10σ case. In Figure 10a, the *LJ* composite, the probability histogram broadens and shifts to the right, indicating that the voids, in general, increase in size with increasing strain. In panel (b), the *interWCA* system, the sharp curve broadens to a flat plateau with increasing strain, indicating the voidspace is composed of a broad distribution of pore sizes which both grow and emerge as strain increases. This quantitative observation agrees with the snapshot shown in Figure 6d, where at ϵ = 1.0, extended material delamination originating at FP–polymer interfaces is present.

**Figure 10:**
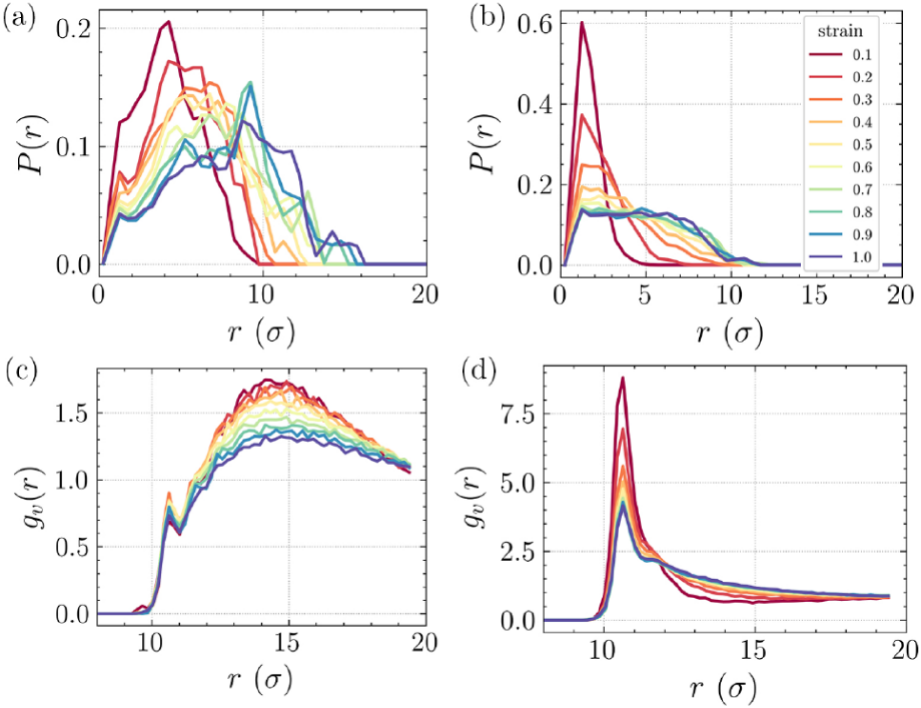
Pore size distributions with increasing engineering strain for the (a) *LJ* and (b) *interWCA* composites and voidspace distribution functions with increasing strain for the (c) *LJ* and (d) *interWCA* composites. All data is for systems with ϕ = 0.5 and *T* = 0.3.

While the sizes of the pores change with increasing ϵ for both cases, the spatial arrangement of these voids stays relatively constant with increasing strain. *g*_*v*_(*r*) curves for the *LJ* composite in Figure 10c show a broad distribution, largely unchanged from the blue curve in Figure 8, which represents the *LJ* system. Similarly panel (d) shows *g*_*v*_(*r*) for the *interWCA* composite, indicating that the voids exist at the FP–polymer interface. The right shoulder broadens with increasing ϵ, capturing the evolution of larger voids that begin at the FP–polymer interface. We emphasize that the magnitude of *g*_*v*_(*r*) at a given *r* encodes the local-to-bulk density ratio of voidspace in the spherical shell at that radius. Cross-condition comparisons of absolute peak amplitude are therefore mediated by the total accessible voidspace in each system, but the position of peaks, the central observable distinguishing interfacial delamination from bulk cavitation, is robust to this normalization.

### Interfacial Polymer Structure and the Temperature Dependence of Failure

To begin to bridge the macroscopic stress–strain response to the microscopic interaction chemistry, we examined the polymer density profile near the filler particles via the radial distribution function *g*_FP–polymer_(*r*). Distinct from the voidspace distribution function *g*_*v*_(*r*) introduced earlier, *g*_FP–polymer_(*r*) is the standard radial distribution function between FP centers and polymer beads: it asymptotes to unity at large *r* for a spatially uniform polymer phase, with values above unity indicating enhanced local polymer density relative to bulk and values below unity indicating depletion. Equilibrated configurations at ϕ = 0.25, *r* = 10σ for the four interaction scenarios at both temperatures are presented in Figure 11. This analysis characterizes the equilibrium interfacial structure that precedes any imposed deformation, providing a static structural reference point against which the temperature-dependent design trade-off observed in Figure 4 can be interpreted.

**Figure 11:**
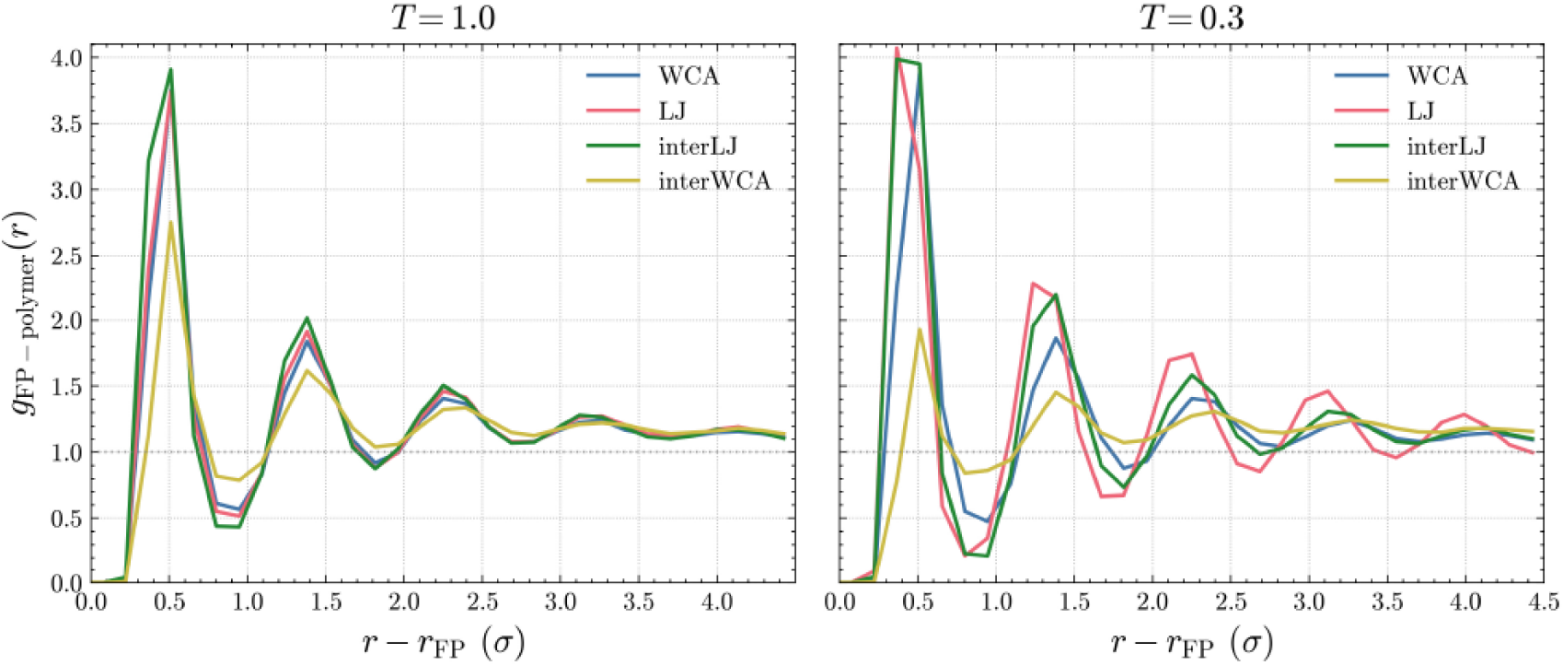
Polymer radial distribution functions *g*_FP–polymer_(*r*) relative to filler particle centers, for the four interaction scenarios at (left) *T* = 1.0 and (right) *T* = 0.3. The *x*-axis is shifted so that the FP outer surface (*r*_FP_ = 10σ) sits at the origin. All curves are taken from equilibrated (prestrain) configurations at ϕ = 0.25, *r* = 10σ. The dotted horizontal line at *g* = 1 indicates the system-wide reference polymer density.

The four scenarios show the same qualitative structure: a first-shell contact peak at *r* – *r*_FP_ ≈ 0.5σ from hard-wall packing of polymer beads against the FP surface, followed by oscillations that decay to the bulk polymer density at *r* – *r*_FP_ ∼ 3σ and beyond. The amplitude of the first-shell contact peak, however, differs systematically across interaction scenarios. At *T* = 1.0, the *WCA*, *LJ*, and *interLJ* scenarios all show a similar contact peak of *g*  ≈  3.7–3.9, while *interWCA*’s contact peak is reduced to *g*  ≈  2.7, indicating ∼30% fewer polymer beads in direct contact with the FP surface. At *T* = 0.3, the contrast strengthens: *WCA*, *LJ*, and *interLJ* contact peaks sharpen to *g*  ≈  4.0, while *interWCA*’s peak drops further to *g*  ≈ 1.95, approximately half the magnitude of the other three. Oscillation amplitudes throughout the first ∼2σ outside the FP surface are likewise damped for *interWCA* relative to the others.

This systematic suppression of the interfacial polymer density for *interWCA* reflects the absence of favorable polymer–FP cross-interaction: the repulsive cross-potential combined with attractive bulk polymer interactions leaves the polymer with no energetic preference to populate the FP-adjacent region. The structural distinction is present at both temperatures, but its mechanical consequences appear to differ qualitatively across *T*_*g*_. We propose the following interpretation, while noting that direct dynamic verification (e.g., segmental mobility profiles near the FP interface) is left for future work.

Above *T*_*g*_, the suppressed interfacial polymer density may enable the enhanced mechanical reinforcement seen in Figure 4a at *T* = 1.0. In *LJ* and *interLJ*, the strong polymer–FP contact peak corresponds to an interfacial polymer layer that is plausibly mechanically distinct from bulk polymer: the polymer beads in this layer are energetically pinned to the FP surface and would be expected to relax on time scales longer than the strain-rate-defined window. Under tensile load, stress would then concentrate at these pinned interfaces and be transmitted through a quasi-solid bound layer. In *interWCA*, the suppressed contact peak suggests polymer that retains more nearly bulk-like dynamics in the FP-adjacent region. Stress under load would then distribute more uniformly through the polymer matrix rather than concentrating at pinned interfaces, consistent with the enhanced reinforcement of *interWCA* over the other scenarios in the rubbery regime.

Below *T*_*g*_, the same structural feature appears to become a defect rather than a feature. Bulk polymer mobility is arrested; the distribution mechanism described above would no longer be available. The weakened polymer–FP coupling then means simply that there are fewer (and less strongly bound) polymer–FP contacts to resist interfacial separation. With no dynamic recovery, applied stress localizes at the weakest connection, which in *interWCA* is the FP–polymer interface itself. Delamination at the interface becomes the lowest-energy failure mode, consistent with the void localization at the FP surface seen in *g*_*v*_(*r*) (Figure 8) and the degraded reinforcement at *T* = 0.3 in Figure 4b.

In this interpretation, the same chemical passivation (repulsive polymer–FP cross-interaction) produces opposite mechanical outcomes through two distinct mechanical regimes operating on the same underlying interfacial structure: above *T*_*g*_, the weakened interfacial coupling may enable stress redistribution that enhances reinforcement; below *T*_*g*_, the weakened coupling becomes a failure mode because the dynamic recovery that would tolerate it is unavailable. The interfacial polymer density profiles of Figure 11 provide a static structural feature consistent with this temperature-dependent design trade-off, though direct measurement of the implicated dynamic gradients would be a worthwhile extension of this work.

## Conclusions

We implemented the well-traveled Kremer-Grest bead-spring representation of a polymer system to simulate model polymer–filler composites. Varying the interaction potentials enables a unique and rapid exploration of design phase space that allows researchers to rapidly survey the impact of high-level design choices, such as the interaction strength between filler material and bulk polymer. Further, these simulations circumvent the costly *formulate-synthesize-cure-test* benchtop development cycle and, due to the generality of the model, decouple the design of these materials from the selection of backbone and functional group chemistry. This high-level approach to polymer interrogation separates the design from limitations of standard feedstock and synthetic technique and, when a promising candidate is found, can encourage polymer scientists to seek out methods to realize these designs.

Varying FP packing fraction and diameter affect the mechanical enhancement provided by the filler. Contact network analysis of these configurations shows that at high ϕ the filler particles consolidate into a single compact agglomerate containing nearly all of the FP, with mean coordination number well below the isostatic threshold for frictionless sphere jamming. This subisostatic but near-percolating structure rearranges cooperatively during deformation and accounts for the discontinuous stress–strain response observed at ϕ = 0.5.

In the glassy state, voids or delaminations may emerge during deformation of the polymer composite. Analysis of the pore sizes revealed distinct differences as a function of the polymer–FP interaction potential: When the particles and polymer interact with the same attractive potential, the voids formed are significantly larger than when the polymer is not attracted to the FP. To identify the location of these voids relative to the morphology of the composite, we introduced the void distribution function *g*_*v*_(*r*), which combines a controlled insertion of dummy particles with analysis by pair distribution function. This approach provides a spatial quantification of voidspace relative to the filler particles and shows that, in the case of passivated FP, delamination is much more likely to occur at the polymer–FP interface than within the glassy polymer matrix.

Within the parameter ranges examined in this work, the *interWCA* scenario, which models a chemically passivated FP surface in a cohesive polymer matrix, provides enhanced mechanical reinforcement above the polymer *T*_*g*_ relative to the homogeneous attractive (*LJ*) scenario. Below *T*_*g*_, however, the same interaction scheme produces widespread FP–polymer delamination and diminished mechanical reinforcement, with the reinforcement deficit increasing with increasing ϕ: an inversion of the trend observed in systems with homogeneous interparticle potentials. Whether this temperature-dependent design trade-off persists across the broader composite design space (chain length, FP size, interaction strength, strain rate) is an open question that would benefit from focused follow-up studies.

Quantitative features of the glassy-regime results presented here are expected to depend on strain rate when compared to the quasi-static limit. Within the strain-rate range accessible to us for a representative *LJ* configuration, however, the void localization trends remain insensitive to *ϵ̇*, consistent with their origin in features of the relaxed starting configuration rather than in rate-dependent cavitation kinetics. We did not test rate-sensitivity for all systems and parameter combinations due to computational constraints; whether the rate-robustness observed here generalizes to those cases is left for future work.

Pore size distributions evolve differently for the low-temperature *LJ* and *interWCA* systems but the spatial position of the voids, relative to the filler particles, remains constant with increasing strain for both systems and suggests that the failure mechanism in each case does not vary with strain.

## Supplementary Material



## Notes

A detailed description of replicate sampling, contact network analysis, *g*_*v*_(*r*) calculation and validation, and tests at other strain rates (PDF)

## References

[1] Staudinger H. (1920). Über
Polymerisation. Ber. Dtsch. Chem. Ges. A/B.

[2] Mülhaupt R. (2004). Hermann Staudinger
and the Origin of Macromolecular Chemistry. Angew. Chem., Int. Ed.

[3] Rubinstein, M. Polymer Physics. Oxford University Press, 2003.

[4] Tillotson T., Hrubesh L. (1992). Transparent Ultralow-Density
Silica Aerogels Prepared
by a Two-Step Sol-Gel Process. J. Non-Cryst.
Solids.

[5] Pekala R. W., Alviso C. T. (1992). Carbon Aerogels and Xerogels. MRS Online Proc. Libr.

[6] Pekala R. W., Schaefer D. W. (1993). Structure of Organic
Aerogels. 1. Morphology and Scaling. Macromolecules.

[7] Witten T. A., Rubinstein M., Colby R. H. (1993). Reinforcement of Rubber by Fractal
Aggregates. J. Phys. II.

[8] Yadav R., Singh M., Shekhawat D., Lee S.-Y., Park S.-J. (2023). The Role
of Fillers to Enhance the Mechanical, Thermal, and Wear Characteristics
of Polymer Composite Materials: A Review. Composites,
Part A.

[9] Jouault N., Vallat P., Dalmas F., Said S., Jestin J., Boué F. (2009). Well-Dispersed
Fractal Aggregates as Filler in Polymer-Silica
Nanocomposites: Long-Range Effects in Rheology. Macromolecules.

[10] Maiti A., Small W., Gee R. H., Weisgraber T. H., Chinn S. C., Wilson T. S., Maxwell R. S. (2014). Mullins Effect in
a Filled Elastomer under Uniaxial Tension. Phys.
Rev. E.

[11] Rueda M. M., Auscher M.-C., Fulchiron R., Périé T., Martin G., Sonntag P., Cassagnau P. (2017). Rheology and
Applications of Highly Filled Polymers: A Review of Current Understanding. Prog. Polym. Sci.

[12] Domurath J., Saphiannikova M., Heinrich G. (2017). The Concept of Hydrodynamic Amplification
Infilled Elastomers. Kautsch. Gummi Kunstst.

[13] Fu S.-Y., Feng X.-Q., Lauke B., Mai Y.-W. (2008). Effects of Particle
Size, Particle/Matrix Interface Adhesion and Particle Loading on Mechanical
Properties of Particulate–Polymer Composites. Composites, Part B.

[14] Vacatello M. (2002). Molecular
Arrangements in Polymer-Based Nanocomposites. Macromol. Theory Simul.

[15] Lin E. Y., Frischknecht A. L., Riggleman R. A. (2020). Origin of Mechanical Enhancement
in Polymer Nanoparticle (NP) Composites with Ultrahigh NP Loading. Macromolecules.

[16] Sun R., Melton M., Safaie N., Ferrier R. C., Cheng S., Liu Y., Zuo X., Wang Y. (2021). Molecular View on Mechanical Reinforcement
in Polymer Nanocomposites. Phys. Rev. Lett.

[17] Shi R., Yu L., Zhang N., Yang Y., Lu Z.-Y., Qian H.-J. (2023). Molecular
Origin of the Reinforcement Effect and Its Strain-Rate Dependence
in Polymer Nanocomposite Glass. ACS Macro Lett.

[18] Shen J., Lin X., Liu J., Li X. (2020). Revisiting Stress–Strain Behavior
and Mechanical Reinforcement of Polymer Nanocomposites from Molecular
Dynamics Simulations. Phys. Chem. Chem. Phys.

[19] Ozmusul M. S., Picu C. R., Sternstein S. S., Kumar S. K. (2005). Lattice Monte Carlo
Simulations of Chain Conformations in Polymer Nanocomposites. Macromolecules.

[20] Shui Y., Huang L., Wei C., Chen J., Song L., Sun G., Lu A., Liu D. (2021). Intrinsic Properties of the Matrix
and Interface of Filler Reinforced Silicone Rubber: An in Situ Rheo-SANS
and Constitutive Model Study. Compos. Commun.

[21] Móczó, J. ; Pukánszky, B. Particulate Fillers in Thermoplastics In Fillers for Polymer Applications Springer: Berlin Heidelberg, 2016; pp. 1–43

[22] Lin E. Y., Frischknecht A. L., Riggleman R. A. (2021). Chain and Segmental Dynamics in Polymer–Nanoparticle
Composites with High Nanoparticle Loading. Macromolecules.

[23] Afzal M. A. F., Browning A. R., Goldberg A., Halls M. D., Gavartin J. L., Morisato T., Hughes T. F., Giesen D. J., Goose J. E. (2021). High-Throughput
Molecular Dynamics Simulations and Validation of Thermophysical Properties
of Polymers for Various Applications. ACS Appl.
Polym. Mater.

[24] Bowman A. L., Mun S., Nouranian S., Huddleston B. D., Gwaltney S. R., Baskes M. I., Horstemeyer M. F. (2019). Free Volume and Internal Structural Evolution during
Creep in Model Amorphous Polyethylene by Molecular Dynamics Simulations. Polymer.

[25] Estridge C. E. (2018). The Effects
of Competitive Primary and Secondary Amine Reactivity on the Structural
Evolution and Properties of an Epoxy Thermoset Resin during Cure:
A Molecular Dynamics Study. Polymer.

[26] Karnes J. J., Weisgraber T. H., Oakdale J. S., Mettry M., Shusteff M., Biener J. (2020). On the Network
Topology of Cross-Linked Acrylate Photopolymers:
A Molecular Dynamics Case Study. J. Phys. Chem.
B.

[27] Voth, G. A. Coarse-Graining of Condensed Phase and Biomolecular Systems. CRC Press: Boca Raton, 2008.

[28] Pavlov A.
S., Khalatur P. G. (2016). Fully Atomistic
Molecular Dynamics Simulation of Nanosilica-Filled
Crosslinked Polybutadiene. Chem. Phys. Lett.

[29] Alessandri R., Grünewald F., Marrink S. J. (2021). The Martini Model in Materials Science. Adv. Mater.

[30] Karnes J. J., Weisgraber T. H., Cook C. C., Wang D. N., Crowhurst J. C., Fox C. A., Harris B. S., Oakdale J. S., Faller R., Shusteff M. (2023). Isolating Chemical Reaction Mechanism as a Variable
with Reactive Coarse-Grained Molecular Dynamics: Step-Growth versus
Chain-Growth Polymerization. Macromolecules.

[31] Kremer K., Grest G. S. (1990). Dynamics of Entangled
Linear Polymer Melts: A Molecular-dynamics
Simulation. J. Chem. Phys.

[32] Lin E. Y., Frischknecht A. L., Winey K. I., Riggleman R. A. (2021). Effect
of Surface Properties and Polymer Chain Length on Polymer Adsorption
in Solution. J. Chem. Phys.

[33] Thompson A.
P., Aktulga H. M., Berger R., Bolintineanu D. S., Michael Brown W., Crozier P. S., in’t Veld P. J., Kohlmeyer A., Moore S. G., Nguyen T. D. (2021). LAMMPS
- A Flexible Simulation Tool for Particle-Based Materials Modeling
at the Atomic, Meso, and Continuum Scales. Comput.
Phys. Commun.

[34] Lenhardt, J. M. Llama 20, 40, 50 and 60 Siloxanes for Direct Ink Write – Compositional Information. https://www.osti.gov/biblio/1871381 Accessed 01 April 2026.

[35] Schmidt S.
C., Grondz J. J., Ford M. J., Armas J. A., Alexopoulos A. D., Mendoza J. T., Loeb C. K., Lenhardt J. M. (2025). 3D Printable Silicone
Compositions Exhibiting High Toughness and Low Durometer. J. Mater. Res.

[36] Mohottalalage S. S., Karnes J. J., Schmidt S. C., Weisgraber T. H., Saab A. P., Maiti A. (2026). Structure and Flow-Viscosity of Filled-Polymer
Based 3D Printing Ink: Exploration through Coarse-Grained Molecular
Dynamics. ACS Omega.

[37] Torquato S., Stillinger F. H. (2007). Toward the Jamming Threshold of Sphere
Packings: Tunneled
Crystals. J. Appl. Phys.

[38] Hales, T. C. An overview of the Kepler conjecture. arXiv 2002.

[39] Jain T. S., de Pablo J. J. (2004). Investigation of
Transition States in Bulk and Freestanding
Film Polymer Glasses. Phys. Rev. Lett.

[40] Ford, A. Molecular Dynamics Modeling of Heterogeneous Structure and Rheology of Human Lung Mucus. Ph.D. thesis, University of North Carolina, 2022.

[41] Mermet-Guyennet M. R. B., Gianfelice
de Castro J., Varol H. S., Habibi M., Hosseinkhani B., Martzel N., Sprik R., Denn M. M., Zaccone A., Parekh S. H., Bonn D. (2015). Size-Dependent Reinforcement
of Composite Rubbers. Polymer.

[42] Varol H. S., Meng F., Hosseinkhani B., Malm C., Bonn D., Bonn M., Zaccone A., Parekh S. H. (2017). Nanoparticle Amount,
and Not Size, Determines Chain Alignment and Nonlinear Hardening in
Polymer Nanocomposites. Proc. Natl. Acad. Sci.
U. S. A.

[43] Hagita K., Morita H., Doi M., Takano H. (2016). Coarse-Grained Molecular
Dynamics Simulation of Filled Polymer Nanocomposites under Uniaxial
Elongation. Macromolecules.

[44] Hagita K., Morita H., Takano H. (2016). Molecular
Dynamics Simulation Study
of a Fracture of Filler-Filled Polymer Nanocomposites. Polymer.

[45] Sarkisov L., Bueno-Perez R., Sutharson M., Fairen-Jimenez D. (2020). Materials
Informatics with PoreBlazer v4.0 and the CSD MOF Database. Chem. Mater.

