# Supplementary material for: On Void Formation during the Simulated Tensile Testing of Polymer–Filler Particle Composites
Source: Macromolecules. 2026 Jul 21;59(15):8569–80. doi: 10.1021/acs.macromol.6c00355 (PMC13472198; doi:10.1021/acs.macromol.6c00355)
Supplement: Supplementary file 1 [file ma6c00355_si_001.pdf]

Supporting Information for "On void formation during the simulated tensile testing of polymer-filler particle composites"

John J. Karnes,* Supun S. Mohottalalage, Amitesh Maiti, Andrew P. Saab, and Todd H. Weisgraber
Lawrence Livermore National Laboratory
5000 East Ave., Livermore, CA, 94550, USA
(Dated: June 12, 2026)

I. REPLICATE SAMPLING FOR REPRESENTATIVE STRESS-STRAIN TRENDS

We assessed the robustness of the central stress-strain trends in this work by performing independent replicate simulations for four representative conditions. Three independent starting configurations were prepared per condition using the full system setup, relaxation, and equilibration protocol described in the Methods, with different random seeds during initial filler particle and polymer chain placement. For each configuration, uniaxial extensions were performed along the x, y, and z axes and averaged as described in the main text. $\phi = 0$ configurations (no filler) were only extended in the x-direction; no additional checks for anisotropy. Each curve shown in Figures S1 and S2 therefore represents the mean of nine independent simulations per polymer composite condition, with shaded bands indicating the inter-replicate range.

Figure S1 shows replicate stress-strain curves for three ϕ-sweep panels covering the central interaction-potential contrasts in this work: (a) the canonical reference case (*WCA* at $T = 1.0$, corresponding to main-text Figure 2(a)); (b) the glassy attractive case (*LJ* at $T = 0.3$, corresponding to Figure 2(c)); and (c) the glassy passivated interface case (*interWCA* at $T = 0.3$, corresponding to Figure 2(d), the inverted trend underpinning the design tradeoff discussed in the main text). Figure S2 shows the replicate stress-strain curves for the FP-size sweep in the glassy regime (*LJ* at $T = 0.3$, $\phi = 0.25$, corresponding to Figure 3(c)).

These four conditions were chosen to span the central physical contrasts in this work: the canonical reference, the glassy attractive case, the glassy passivated case with its inverted trend, and the FP-size dependence in the glassy regime.

The qualitative trends reported in the main text are preserved across all replicates: (i) mechanical reinforcement increases monotonically with ϕ under repulsive interactions (Figure S1a); (ii) the *LJ* glassy system exhibits post-yield softening at the highest ϕ, with the $\phi = 0.5$ curve peaking near $\epsilon \approx 0.07$ and declining thereafter (Figure S1b, purple curve), consistent with progressive void accumulation; (iii) the *interWCA* glassy system shows the inverted ϕ-dependence, with mechanical reinforcement decreasing as ϕ increases (Figure S1c); and (iv) in the glassy regime, the no-filler reference is clearly separated from the filled systems while the FP-size dependence among filled systems is subtle but consistent across replicates (Figure S2).

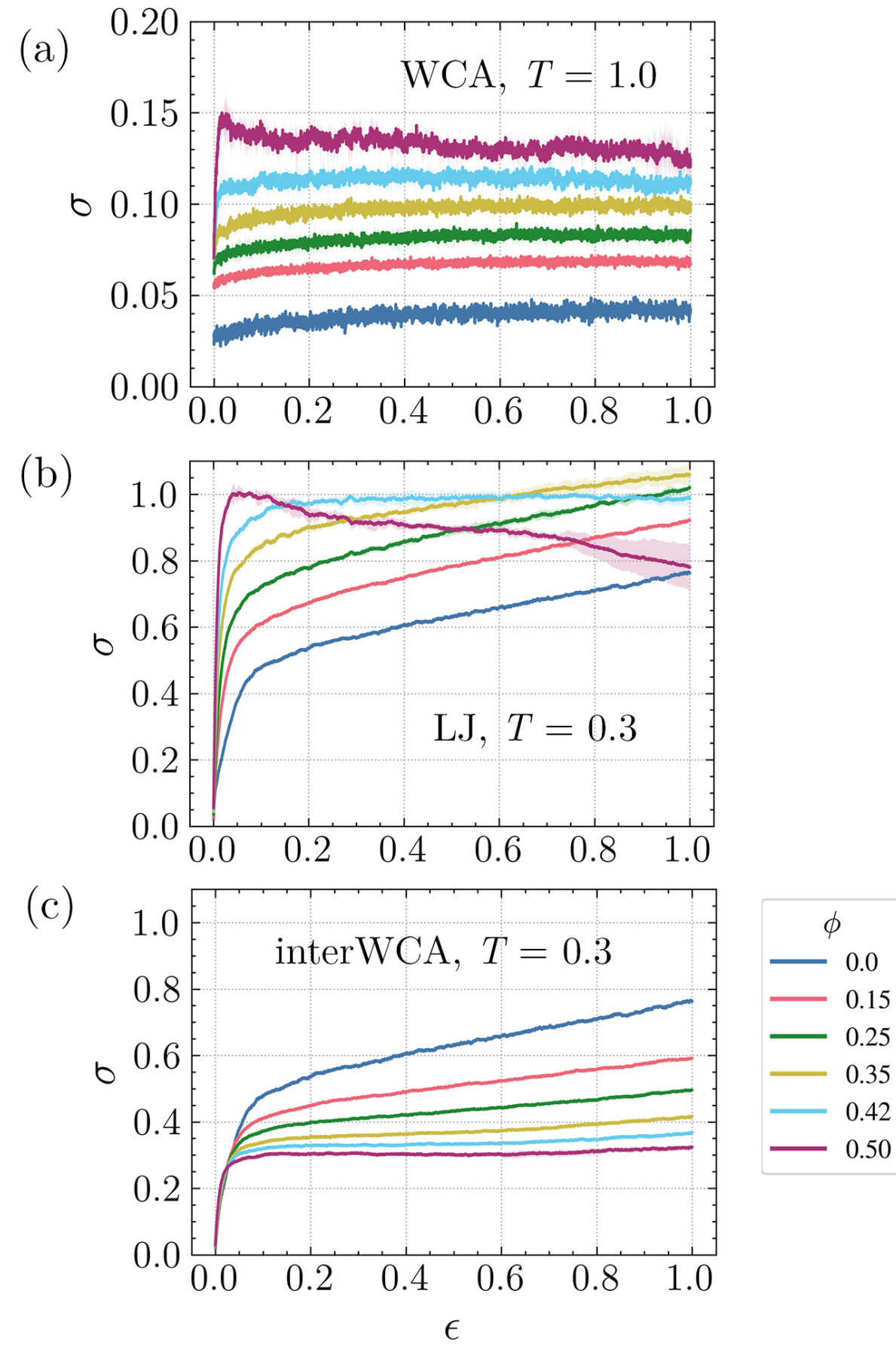

FIG. S1. Replicate stress-strain curves for the ϕ-sweep across three representative interaction-potential conditions. (a) *WCA* at $T = 1.0$, corresponding to main-text Figure 2(a). (b) *LJ* at $T = 0.3$, corresponding to Figure 2(c). (c) *inter-WCA* at $T = 0.3$, corresponding to Figure 2(d). Each curve is the mean of nine independent simulations (three independent starting configurations $\times$ uniaxial extension along x, y, and z); shaded bands indicate the inter-replicate range. Note the post-yield softening at $\phi = 0.5$ in panel (b) and the inverted ϕ-ordering in panel (c).

Inter-replicate variability is small compared to the differences between conditions in all panels. This is ex-

* karnes@llnl.gov

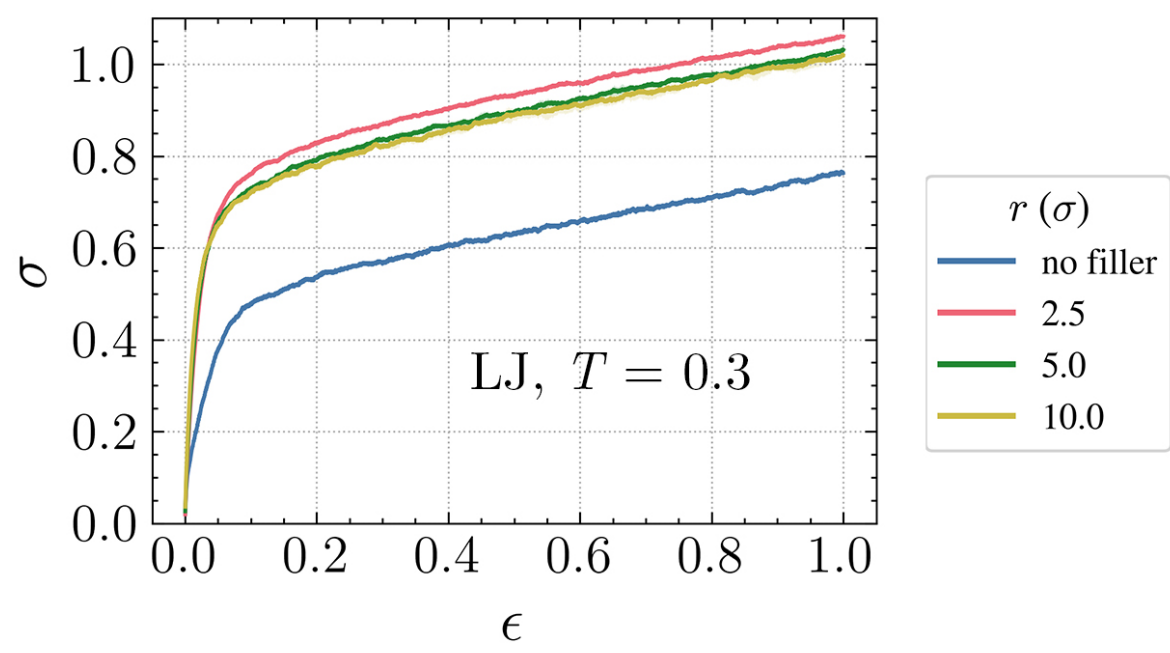

FIG. S2. Replicate stress-strain curves for the FP-size sweep at $\phi = 0.25$ in the glassy regime (LJ at $T = 0.3$), corresponding to main-text Figure 3(c). Each curve is the mean of nine independent simulations (three independent starting configurations × uniaxial extension along x, y, and z); shaded bands indicate the inter-replicate range. The no-filler reference is clearly separated from the three filled systems; differences among the filled systems are subtle but consistent across replicates.

pected given the large simulation cell size used in this work (97.31σ edge length, $\sim 530{,}000$ particles, up to 109 FPs per cell): each cell already contains a large statistical ensemble of microscopic configurations, and inter-cell variance of cell-averaged observables such as stress is correspondingly suppressed.

II. FILLER PARTICLE CONTACT NETWORK ANALYSIS: METHODOLOGY AND FULL RESULTS

A. Network construction and metrics

The contact network is constructed from the FP center positions by defining two particles i and j as connected when $d_{ij} \leq 2R+\delta$, where $R = 10\sigma$ is the FP outer radius and δ is a contact shell thickness. From this network we compute three scalar metrics. The mean coordination number $\bar{z}$ is the average number of neighbors per FP center. The percolation order parameter is

$$S(\phi) = \frac{|\mathcal{C}_{\max}|}{N}, \tag{1}$$

where $|\mathcal{C}_{\max}|$ is the size of the largest connected component and N is the total number of FP centers. The cluster susceptibility analog is

$$\chi(\phi) = \frac{\sum_s{}' s^2 n_s}{\sum_s{}' s\, n_s}, \tag{2}$$

where n_s is the number of clusters of size s and the prime denotes exclusion of the largest cluster. In three-dimensional random percolation universality χ diverges at the critical fill fraction ϕ_c as $\chi \sim |\phi - \phi_c|^{-\gamma}$ with $\gamma \approx 1.80$, providing a sharper locator of the transition than S alone. In a finite simulation cell the divergence is rounded into a peak whose position approximates ϕ_c.

B. Periodic boundary handling

Periodic boundary conditions require careful treatment in percolation analysis: a naive minimum-image distance calculation correctly identifies contacts but cannot distinguish between a finite cluster and one that wraps across the simulation cell. We address this by recording an integer crossing vector $\mathbf{n}_{ij} \in \{-1, 0, +1\}^3$ for each bond, indicating how many box-lengths the bond traverses in each Cartesian dimension. Spanning percolation in direction d is then detected via breadth-first traversal of each connected component while accumulating image offsets: a cluster percolates in direction d if and only if it contains a cycle with nonzero net winding number in that dimension.

C. Choice of contact threshold

The contact shell thickness δ is examined at three physically motivated values. The first, $\delta = 1.12\sigma$, corresponds to the LJ potential minimum at $2^{1/6}\sigma$ beyond geometric surface contact and represents a conservative geometric contact criterion. The second, $\delta = 1.75\sigma$, equals the full LJ interaction cutoff used in the attractive simulations. The third, $\delta = 5.0\sigma \approx R_g$, encompasses the polymer chain radius of gyration and captures FP pairs that can be bridged by a single polymer strand—the polymer-mediated coupling mechanism proposed to underlie reinforcement in filled systems.[1, 2] The location of the χ peak ($\phi_c \approx 0.35$) is found to be insensitive to δ within the range $[1.12, 1.75]\sigma$, shifting modestly to $\phi_c \approx 0.25$ only at $\delta = 5.0\sigma$.

D. Results

Figure S3 summarizes the contact network metrics across the ϕ-series for the WCA and LJ potentials at $T = 1.0$ and $\delta = 1.75\sigma$, showing both the equilibrated (pre-deformation) and 100% uniaxial strain configurations. The order parameter S rises sharply between $\phi = 0.25$ and $\phi = 0.35$ for all conditions and approaches unity at $\phi \geq 0.425$, indicating near-complete consolidation of FPs into a single connected cluster. The susceptibility χ peaks near $\phi_c \approx 0.35$, locating the geometric percolation threshold; the peak is dramatically more pronounced in the LJ 100% strain curve than in the equilibrated LJ curve, indicating that uniaxial extension drives substantial cluster restructuring near ϕ_c. The WCA curves show qualitatively similar but weaker peaks, consistent with the absence of attractive interactions that would otherwise stabilize larger pre-formed agglomerates.

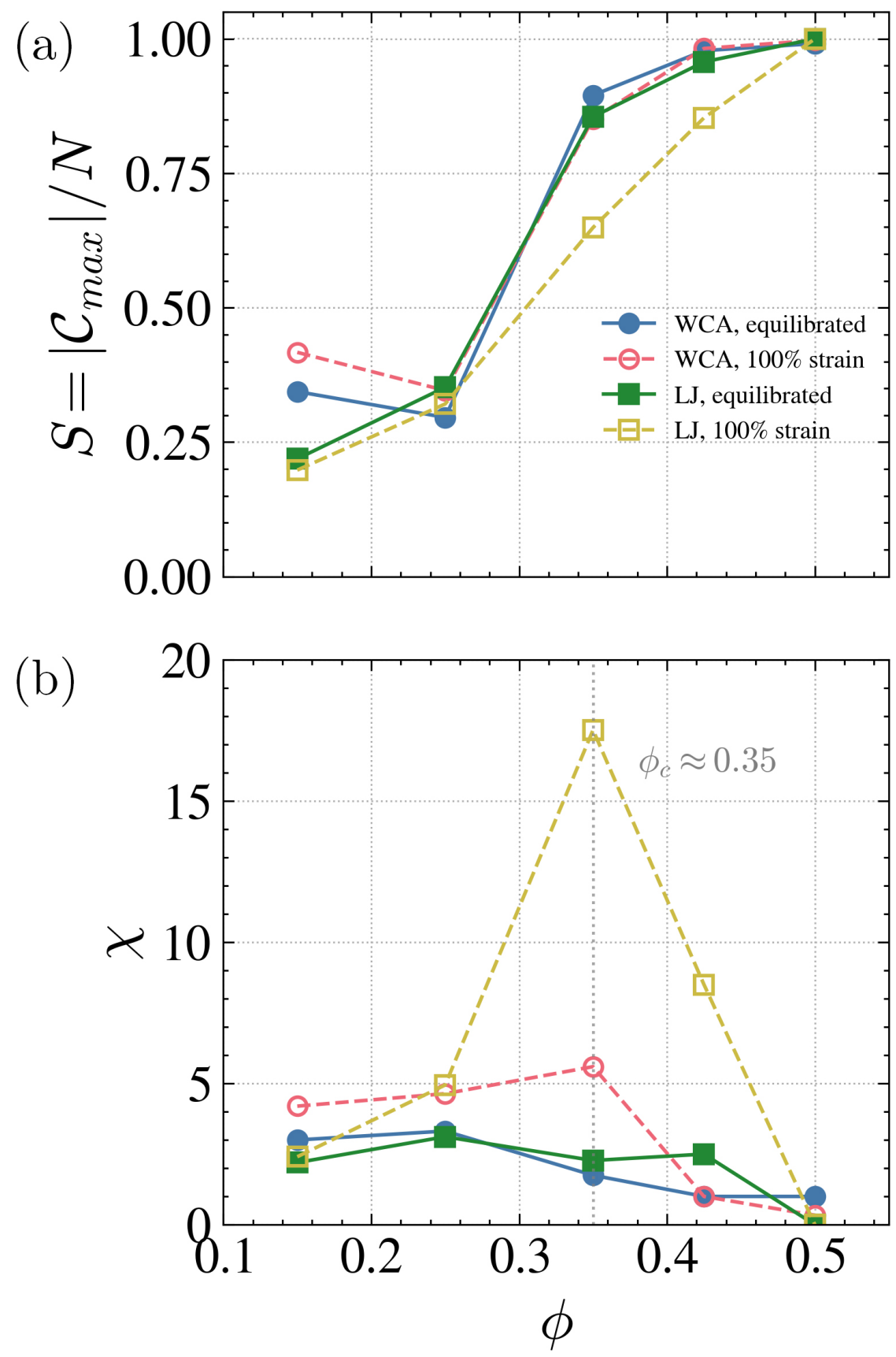

FIG. S3. Contact network metrics across the ϕ-series at $\delta = 1.75\sigma$ for $r = 10\sigma$ filler particles at $T = 1.0$. (a) Percolation order parameter $S = |\mathcal{C}_{\max}|/N$ showing rapid consolidation of FPs into a single connected cluster above $\phi \approx 0.35$. (b) Cluster susceptibility analog χ exhibiting a peak near $\phi_c \approx 0.35$ (vertical dotted line), the geometric percolation threshold. Filled symbols denote equilibrated (pre-deformation) configurations; open symbols denote 100% uniaxial strain configurations averaged over independent extensions along the X, Y, and Z directions. The pronounced χ peak in the LJ post-deformation curve indicates that uniaxial extension drives substantial cluster restructuring near ϕ_c, consistent with the agglomerate breakup discussed in the main text.

Table I provides the full numerical results across all three δ values for completeness. No spanning percolation is observed at any condition, consistent with the compact agglomeration geometry documented in Figure 3(d) of the main text.

TABLE I. Contact network metrics for $r = 10\sigma$ FP, $T = 1.0$. z is the mean coordination number, S the largest-cluster fraction, χ the cluster susceptibility analog. "Pre" denotes relaxed configurations before any uniaxial extension; "Post" denotes 100% uniaxial strain averaged over $x/y/z$ elongation. No spanning percolation was observed at any condition.

		$\delta = 1.12\sigma$			$\delta = 1.75\sigma$			$\delta = 5.0\sigma$	
ϕ	state	z	S	χ	z	S	χ	z	S
**WCA potential**									
0.150	pre	1.25	0.31	2.91	1.38	0.34	3.00	1.75	0.34
0.150	post	1.54	0.35	3.59	1.69	0.42	4.20	2.06	0.53
0.250	pre	1.33	0.30	3.26	1.52	0.30	3.32	2.30	0.50
0.250	post	1.59	0.30	3.73	1.74	0.35	4.64	2.40	0.61
0.350	pre	2.24	0.87	1.80	2.42	0.90	1.75	3.45	0.92
0.350	post	2.60	0.70	11.14	2.83	0.85	5.60	3.53	0.98
0.425	pre	3.03	0.97	1.00	3.38	0.98	1.00	4.62	0.98
0.425	post	3.10	0.97	1.22	3.37	0.98	1.00	4.57	0.99
0.500	pre	3.63	0.99	1.00	3.85	0.99	1.00	5.34	0.99
0.500	post	3.46	0.99	1.00	3.79	1.00	0.33	5.10	1.00
**LJ potential**									
0.150	pre	1.00	0.22	2.20	1.13	0.22	2.20	1.38	0.22
0.150	post	1.04	0.18	2.11	1.21	0.20	2.41	1.58	0.34
0.250	pre	1.33	0.32	3.00	1.48	0.35	3.11	2.30	0.93
0.250	post	1.43	0.28	3.71	1.61	0.32	4.95	2.28	0.48
0.350	pre	2.29	0.86	2.27	2.50	0.86	2.27	3.66	0.96
0.350	post	2.77	0.64	16.51	3.02	0.65	17.52	3.96	0.97
0.425	pre	3.14	0.94	2.00	3.40	0.96	2.50	4.50	1.00
0.425	post	2.88	0.80	8.26	3.12	0.85	8.50	4.29	0.99
0.500	pre	3.74	1.00	0.00	3.96	1.00	0.00	5.34	1.00
0.500	post	3.47	0.98	0.83	3.86	1.00	0.00	5.18	1.00

III. CALCULATING VOIDSPACE DISTRIBUTION FUNCTIONS

In the main text we adapt the well-known pair distribution function, $g(r)$, to enable spatial quantification of voids or delaminations that form during the simulated tensile testing of polymer composites. In this section we outline the modification of simulation snapshots to enable the calculation of the voidspace distribution function $g_v(r)$. This overview refers to operations within the LAMMPS environment [3, 4] and we refer the interested reader to LAMMPS documentation for additional details regarding the individual commands mentioned. Figure S4 is a visual outline of the steps described below using a 2-D slice of an elongated simulation cell for clarity.

A. Filling the voids

Our approach involves filling the appropriate void spaces with a unique particle type ('dummy atoms') that will permit calculation of $g_v(r)$. We first read in a simulation snapshot and allow for two new atom types,

```
read_data dataOut.data extra/atom/types 2
```

and insert new dummy atoms by looping over a command such as

```
create_atoms 5 random 10000 121717 //
NULL overlap 1.05 maxtry 10000
```

This action will fill all of the simulation voidspaces with dummy atoms of type 5 as in Figure S4(b). However, since we represent the filler particles (FP) as hollow shells (i.e. Fibonacci spheres), the insides of the FP will also be filled with dummy atoms.

B. Clean out the filler particles

Dummy atoms within the filler particles will make calculation of $g_v(r)$ difficult since not only will the reference atom at the center of each FP be surrounded by dummy atoms, the reference atom is also likely to 'see' dummy particles within nearby FP. Adjusting the range of $g_v(r)$ is not sufficient. We use the LAMMPS command `fix bond/react` [5, 6] to mutate dummy atoms inside the FP into an atom type that will be ignore by the calculation of $g_v(r)$, in this example `type 6`.

```
fix fbr all bond/react stabilization yes //
nvt_grp 0.01 react myrxn cleanup //
1 1.0 ${FP_rad} mol1 mol2 map.txt //
stabilize_steps 0
```

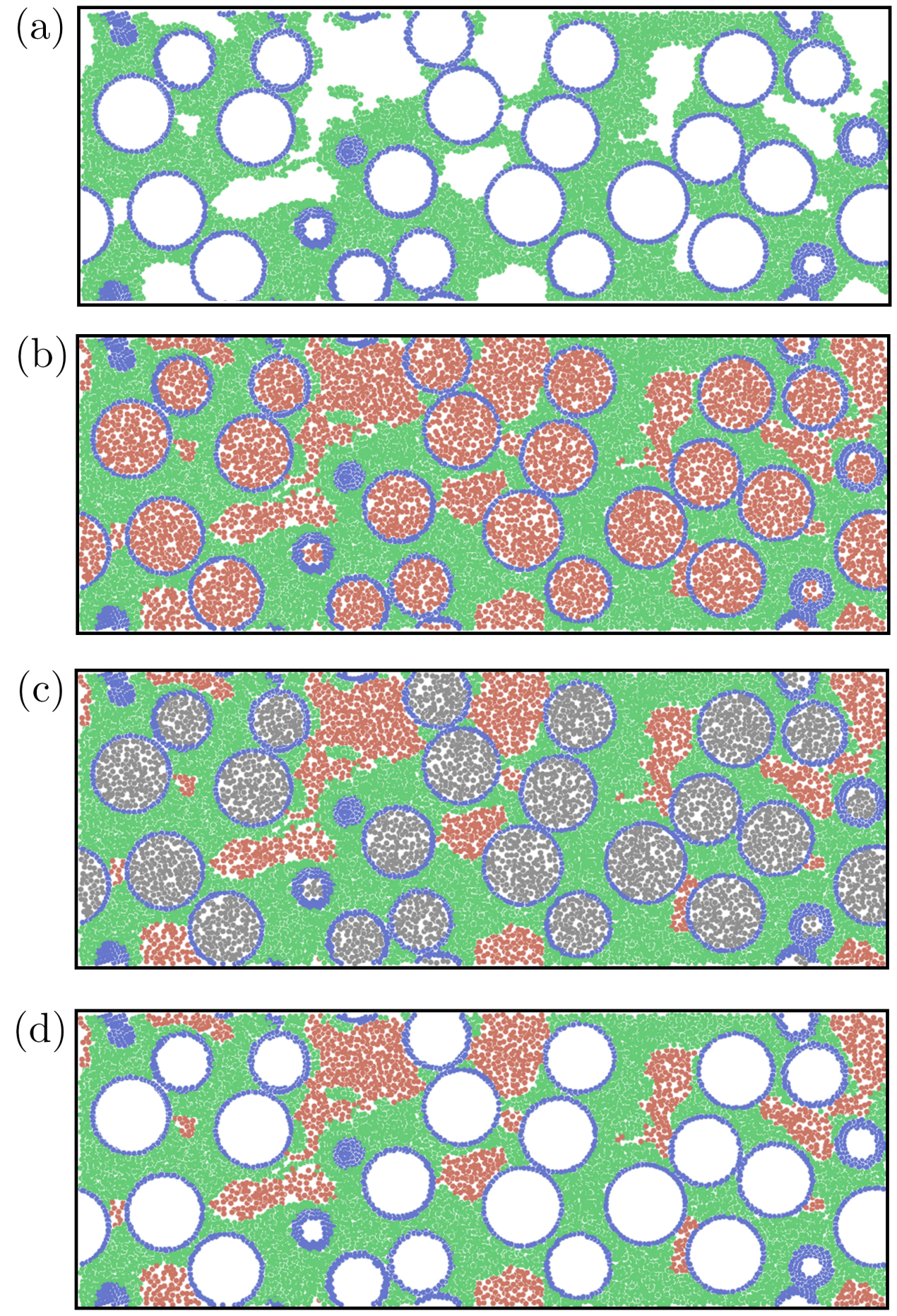

FIG. S4. Visual outline of the snapshot preparation for $g_v(r)$ calculation. (a) Snapshot from simulated uniaxial extension (b) insertion of dummy atoms (c) mutation of dummy atoms within FP (d) mutated dummy atoms made invisible for visual clarity.

C. Compute $g_v(r)$

The modified snapshot (Figure S4(c)) now permits calculation of conventional $g(r)$, such as the proximity of FP to other FP or spatial quantification of voidspace by referencing the dummy atoms inserted by the preceding approach. For example,

```
compute rdfs all rdf 100 1 1 3*4 5 //
cutoff 19.5
fix prdf all ave/time 1 1 1 //
c_rdfs[*] file rdf.txt mode vector
```

The above command calculates several $g(r)$, including $g_{\text{FP-FP}}(r)$, $g_{\text{FP-polymer}}(r)$, and $g_v(r)$ with the centers of the FP used as reference atoms.

IV. VALIDATION OF $g_v(r)$ ON A CONTROLLED GEOMETRY

The void-particle insertion procedure described in SI Section III places dummy particles uniformly throughout the simulation cell, including inside the hollow interiors of the rigid Fibonacci sphere filler particles. The `fix bond/react` step subsequently relabels the FP-interior particles as a distinct LAMMPS type ('dummy particles,' type 6 in this work) so that they do not contribute to the $g_v(r)$ calculation for the polymer voidspace ('voidspace particles,' type 5). The type-6 particles are ignored by $g_v(r)$ but provide a controlled geometry with which to validate the $g_v(r)$ methodology: they capture our attempt to uniformly distribute void/dummy particles within spheres of known radius ($r_{\mathrm{FP}} = 10\sigma$ in the present example) centered on the FP centers, and their radial distribution function relative to those centers has a predictable structure that can be compared to the observed $g(r)$.

Figure S5 shows $g_{1,6}(r)$ for the $\phi = 0.5$, $r = 10\sigma$, *LJ* interaction scheme, computed via the same LAMMPS `compute rdf` used to produce the $g_v(r)$ figures in the main text. The curve exhibits three expected features:

- $g(r)$ is zero for $r < 1\sigma$ (the center bead's exclusion shell) and for $r > r_{\mathrm{FP}} = 10\sigma$ (no type-6 particles outside FPs).
- Sharp peaks at $r \approx 1\sigma$ and $r \approx 8.5\sigma$ correspond to random-sequential-addition packing against the inner boundary (the center bead) and the outer boundary (the inner surface of the Fibonacci sphere), respectively.
- The bulk of the FP interior shows nearly uniform packing structure, consistent with the random sequential addition procedure used by LAMMPS `create_atoms`.

These features demonstrate that the LAMMPS `rdf` compute correctly resolves the spatial structure of a known particle distribution and that the standard pair distribution function normalization is consistently applied. The $g_v(r)$ results reported in the main text, computed using the same code path on the type-5 polymer-voidspace particles, inherit this normalization.

A. Insertion uniformity across FPs

To verify that the void-particle insertion saturates uniformly across all FPs in the simulation, each type-6 particle was assigned to its nearest FP center geometrically (with periodic boundary conditions). Figure S6 shows the resulting distribution of type-6 particle counts per FP across the 93 FPs in the test configuration: 1597 ± 11 particles per FP (range 1574–1618), with all particles assigned at distances less than r_{FP} from their nearest FP center. The corresponding packing density inside the FPs is $0.445 \pm 0.003\ \sigma^{-3}$, with a relative standard deviation of 0.7% across all FPs. This uniformity indicates that the insertion procedure is saturated and that any FP-to-FP variation in the polymer voidspace insertion (type 5) is not introduced by the insertion algorithm itself but reflects genuine differences in the accessible polymer voidspace around each FP.

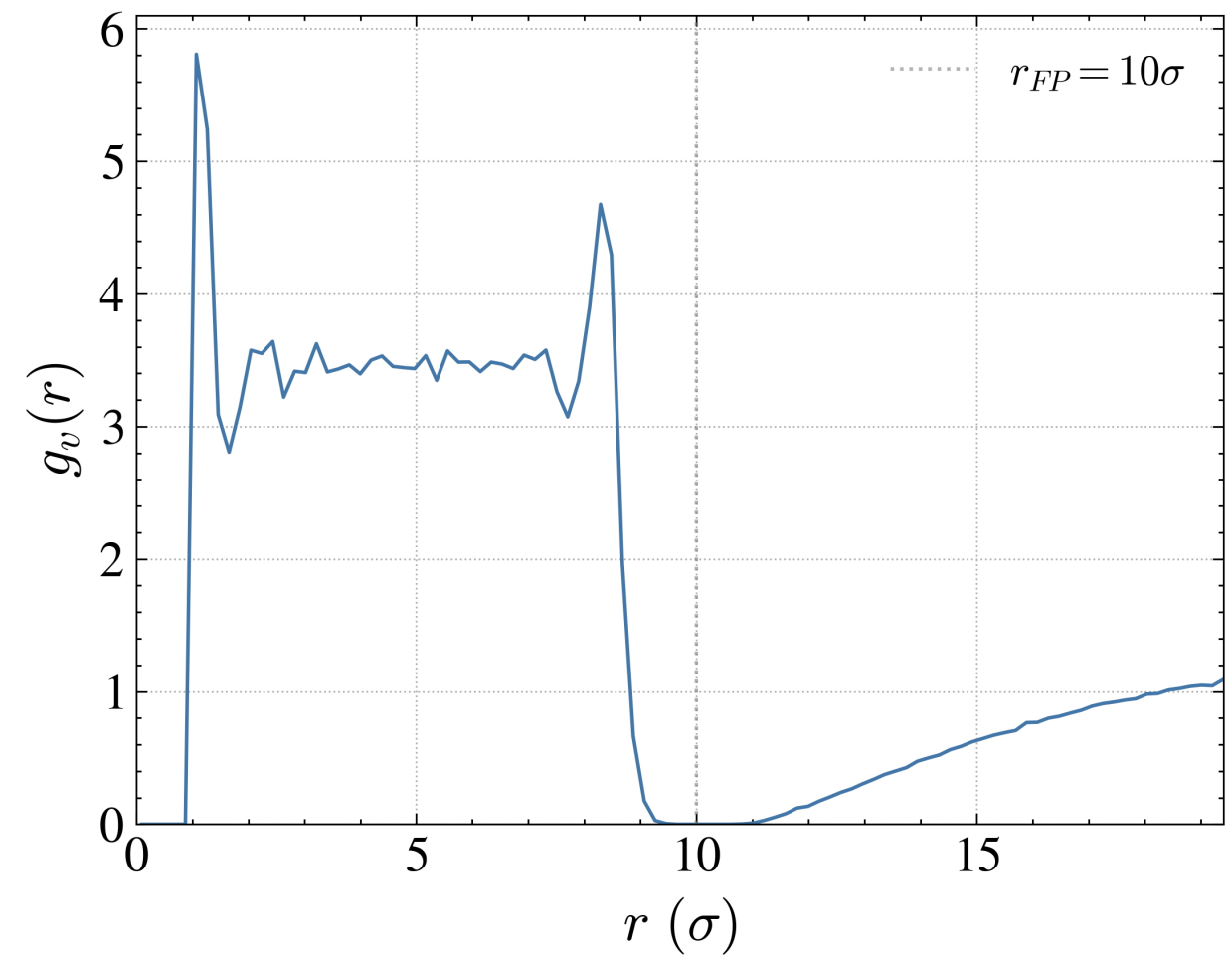

FIG. S5. Radial distribution function $g_{1,6}(r)$ of FP-interior dummy particles (type 6) relative to FP centers (type 1), for the $\phi = 0.5$, $r = 10\sigma$, LJ configuration. The sharp boundary peaks at $r \approx 1\sigma$ and $r \approx 8.5\sigma$ correspond to packing against the FP center bead and the inner Fibonacci-sphere surface, respectively. $g(r) \approx 0$ for $r > r_{\mathrm{FP}} = 10\sigma$ as expected.

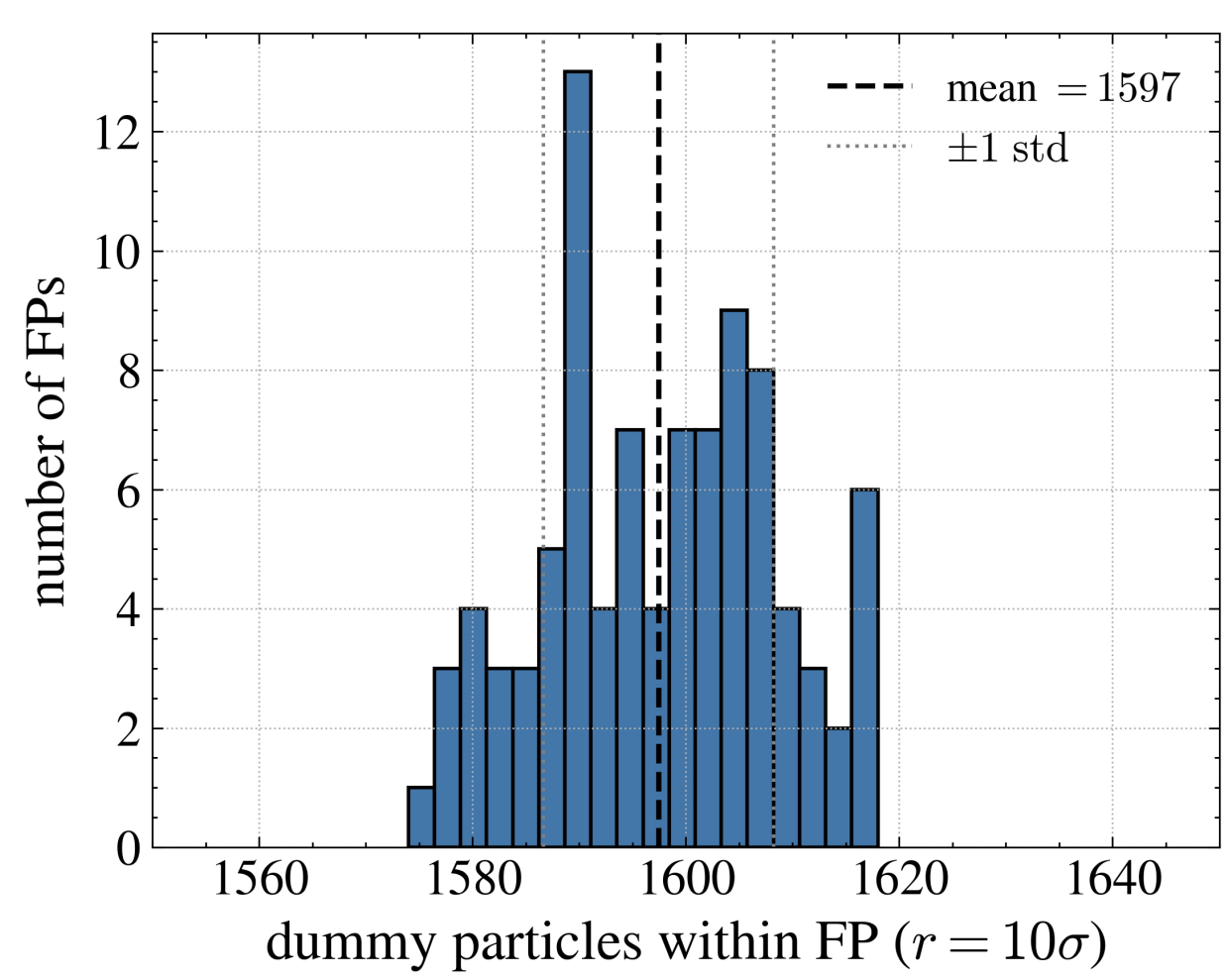

FIG. S6. Distribution of FP-interior dummy particle counts across the 93 FPs in the test configuration. The mean is 1597±11 particles per FP with a relative standard deviation of 0.7%, indicating uniform saturation of the insertion procedure across all FPs regardless of position in the cell.

V. STRAIN RATE IN $T < T_g$ SIMULATIONS

As discussed in the main text, quasistatic uniaxial extension of simulation cells in the glassy regime is beyond the reach of contemporary computational resources when implementing the level of theory in this work. We did perform simulations to probe the sensitivity to strain rate, $\dot{\epsilon}$, increasing and decreasing $\dot{\epsilon}$ by a factor of 2. Figure S7 shows stress-strain curves for the $\phi = 0.425$, $T = 0.3$, $r = 10\sigma$ configuration with the LJ interaction potential applied. For reference, $\dot{\epsilon_0}$ is the strain rate used in the main text, 2×10^{-5}.

The three curves overlay well enough that the stress-strain data obtained at any of these rates could be swapped into Figure 2c of the main text for the $\phi = 0.425$ curve without affecting our analysis or discussion. Stress data obtained at the faster rate, $2 \times \dot{\epsilon_0}$, shows slightly higher stress at $\epsilon < 0.5$, with a maximum deviation of approximately 4% relative to the baseline strain rate, consistent with mildly increased dissipation at the faster rate.

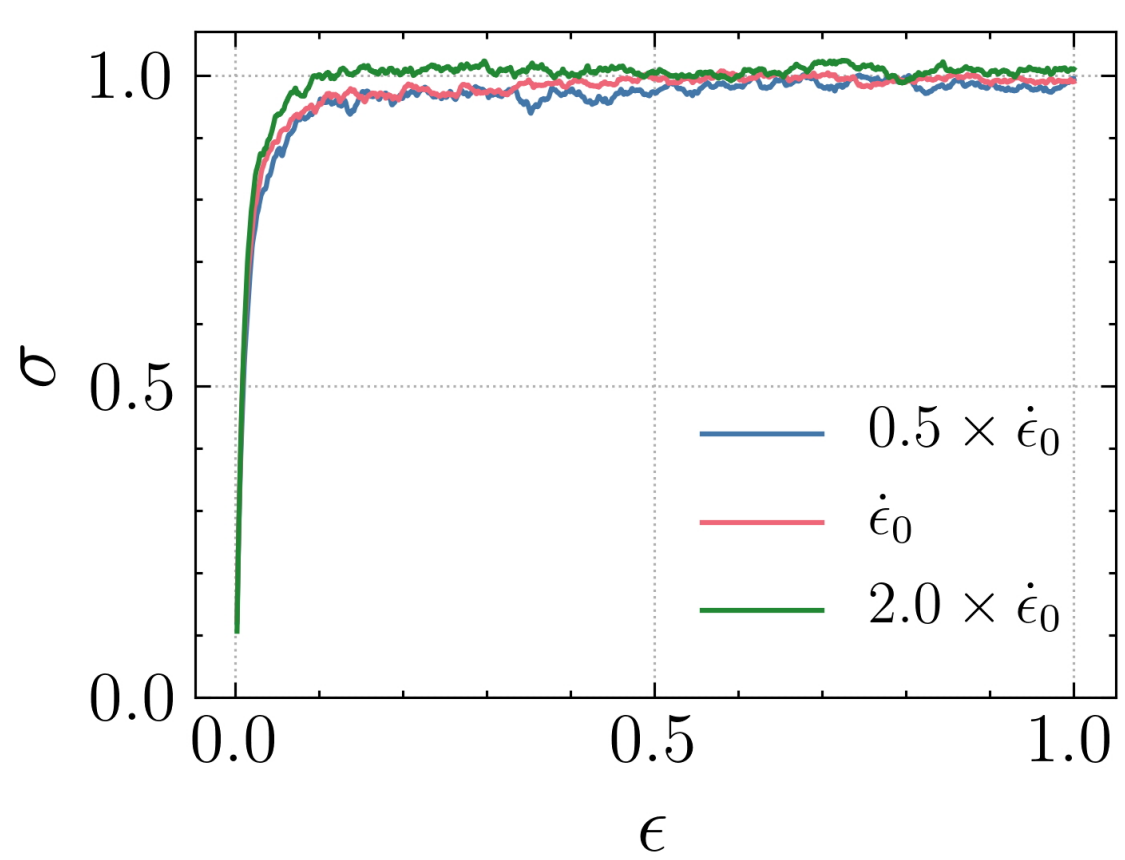

FIG. S7. Stress-strain curves from simulated tensile testing. The three curves represent calculations using an identical, relaxed starting configuration tested at three different strain rates.

A detailed study of strain rates and their effects on void or delamination is beyond the scope of this study and its accessible computational resources. We do include representative snapshots taken from the rate tests above for the interested reader. Figure S8 shows three simulation snapshots: slices taken at $\epsilon = 1.0$ along the axis of elongation. These three uniaxial extension simulations used the same starting configuration and the slices were taken by reducing the cells to a slab 1.15σ wide in y. Visual inspection of the snapshots indicates that void locations are largely repeatable across the three strain rates despite the configurational drift expected from independent runs, suggesting that microscopic configurational precursors in the starting configuration may bias void nucleation toward specific locations, a feature worthy of dedicated future investigation.

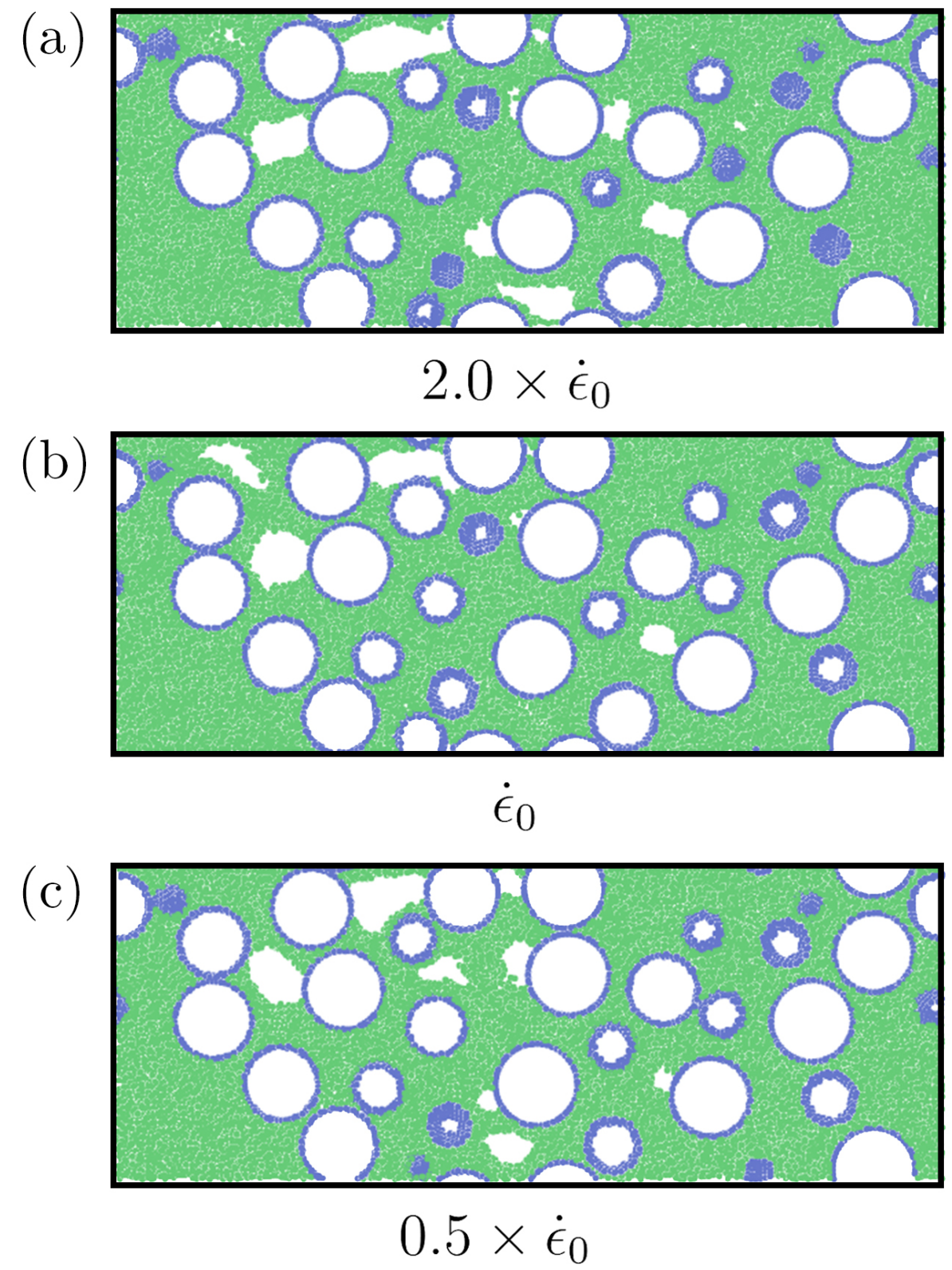

FIG. S8. Simulation snapshots, 'slices' obtained after simulated tensile testing of the same starting configuration at three different strain rates.

VI. POLYMER DENSITY PROFILES NEAR FILLER PARTICLES

To characterize the equilibrium interfacial structure underlying the temperature-dependent design trade-off, we computed the radial distribution function $g_{\mathrm{FP-polymer}}(r)$ between FP centers (atom type 1) and polymer beads (atom types 3 and 4) for the four interaction scenarios at both temperatures. Analysis was performed on equilibrated (pre-strain) configurations at $\phi = 0.25$, $r = 10\sigma$ using the LAMMPS `compute rdf` command with 100 bins over a 14.5σ cutoff. The bin width of $\sim 0.145\sigma$ resolves the first-shell contact features cleanly. Configurations were taken at the end of the equilibration protocol described in the main text, prior to any imposed uniaxial extension.

The radial distribution function returned by LAMMPS asymptotes to 1 at large r for spatially uniform reference particle distributions, with the standard shell-volume factor and reference density included implicitly, as in our mathematically-related calculations of $g_v(r)$. Values of $g_{\mathrm{FP-polymer}}(r) > 1$ in Figure 11 therefore correspond to an enhanced local polymer density at distance r from FP centers; values < 1 correspond to a depletion of polymer density relative to bulk.

[1] M. S. Ozmusul, C. R. Picu, S. S. Sternstein, and S. K. Kumar, Lattice Monte Carlo Simulations of Chain Conformations in Polymer Nanocomposites, Macromolecules **38**, 4495 (2005).

[2] E. Y. Lin, A. L. Frischknecht, and R. A. Riggleman, Origin of Mechanical Enhancement in Polymer Nanoparticle (NP) Composites with Ultrahigh NP Loading, Macromolecules **53**, 2976 (2020).

[3] S. Plimpton, Fast Parallel Algorithms for Short-Range Molecular Dynamics, Journal of Computational Physics **117**, 1 (1995).

[4] A. P. Thompson, H. M. Aktulga, R. Berger, D. S. Bolintineanu, W. Michael Brown, P. S. Crozier, P. J. in 't Veld, A. Kohlmeyer, S. G. Moore, T. D. Nguyen, R. Shan, M. Stevens, J. Tranchida, C. Trott, and S. J. Plimpton, LAMMPS - A flexible simulation tool for particle-based materials modeling at the atomic, meso, and continuum scales, Computer Physics Communications , 108171 (2021).

[5] J. R. Gissinger, B. D. Jensen, and K. E. Wise, Modeling chemical reactions in classical molecular dynamics simulations, Polymer **128**, 211 (2017).

[6] J. R. Gissinger, B. D. Jensen, and K. E. Wise, REACTER: A Heuristic Method for Reactive Molecular Dynamics, Macromolecules **53**, 9953 (2020).
